# Crystal structures reveal transient PERK luminal domain tetramerization in endoplasmic reticulum stress signaling

**DOI:** 10.15252/embj.201489183

**Published:** 2015-04-29

**Authors:** Marta Carrara, Filippo Prischi, Piotr R Nowak, Maruf MU Ali

**Affiliations:** Department of Life Sciences, Imperial CollegeLondon, UK

**Keywords:** cell signaling, ER stress, PERK, unfolded protein response

## Abstract

Stress caused by accumulation of misfolded proteins within the endoplasmic reticulum (ER) elicits a cellular unfolded protein response (UPR) aimed at maintaining protein-folding capacity. PERK, a key upstream component, recognizes ER stress via its luminal sensor/transducer domain, but the molecular events that lead to UPR activation remain unclear. Here, we describe the crystal structures of mammalian PERK luminal domains captured in dimeric state as well as in a novel tetrameric state. Small angle X-ray scattering analysis (SAXS) supports the existence of both crystal structures also in solution. The salient feature of the tetramer interface, a helix swapped between dimers, implies transient association. Moreover, interface mutations that disrupt tetramer formation *in vitro* reduce phosphorylation of PERK and its target eIF2α in cells. These results suggest that transient conversion from dimeric to tetrameric state may be a key regulatory step in UPR activation.

## Introduction

The unfolded protein response is an important cell signaling system that detects the accumulation of misfolded proteins within the endoplasmic reticulum (ER) and carries out a cellular response that attempts to rectify the imbalance. These responses include transcriptional up-regulation of UPR target genes, global cell translation attenuation, and activation of ER-associated degradation pathways. If the imbalance is not rectified, then the UPR switches from being pro-survival to eliciting an apoptotic response (Zhang & Kaufman, 2008; Hetz *et al*, 2011; Walter & Ron, 2011). There are three sensor/transducer proteins: Ire1, PERK, and Atf6, that are critical for initiating mammalian UPR cell signaling and give rise to three separate branches of the response. All three proteins have a luminal sensor/transducer domain that in concert with BiP is vital for sensing ER stress, an ER transmembrane region, and a cytosolic domain that propagates the UPR signal (Bertolotti *et al*, 2000; Schröder & Kaufman, 2005; Ron & Walter, 2007; Zhang & Kaufman, 2008).

Crystal structures of yeast and human Ire1 luminal domains have provided a basis for mechanistic understanding of UPR signal activation, although contrasting interpretations of these structures have given rise to differing views on how this occurs (Credle *et al*, 2005; Zhou *et al*, 2006; Gardner & Walter, 2011; Walter & Ron, 2011; Korennykh & Walter, 2012; Parmar & Schröder, 2012; Carrara *et al*, 2013).

In an attempt to shed new light upon the mechanism of UPR activation and to rationalize the differences between Ire1 luminal domain structures, we determined the crystal structures of PERK luminal domains in two different states: one state is the previously characterized dimer arrangement as seen with Ire1, and the other state is a novel tetramer arrangement. These two states of PERK were captured using human and mouse luminal domain proteins. The tetramer reveals an interface with the salient feature being a helix swapped between dimers that implies a transient association. Using a combination of biophysical and biochemical techniques, we show that both human and mouse PERK luminal domains form dimers and tetramers in solution, similar to that observed within the crystal lattices. Additionally, PERK mutants reduce tetramer formation *in vitro* and reduce PERK and eIf2a phosphorylation in cells. These data suggest that transition from luminal domain dimer to transient tetramer state maybe a key step in UPR activation.

## Results

An optimized human PERK luminal domain construct encompassing residues 105–403 was expressed and purified with cleavable N-terminal His-tag in *E. coli*. This construct was partly identified by sequence and structural similarity to the human Ire1 luminal domain structure (Zhou *et al*, 2006), but was also observed as a cleavage product from purified full-length luminal domain protein minus the signal sequence. Concurrently, we also expressed and purified mouse PERK luminal domain encompassing residues 101–399 based on the human PERK-optimized construct. We were able to obtain crystals for both human and mouse PERK luminal domain proteins that diffracted to around 3.1Å and 3.3Å at station IO2 at Diamond Light Source, UK (Table1). Attempts at molecular replacement to obtain phase information were unsuccessful owning to the relatively low sequence identity between Ire1 and PERK luminal domains of 18%. To overcome this, we used a tungsten-derivatized multi-anomalous dispersion (MAD) dataset which yielded a good quality electron density map, from which we were able to build the structure of human PERK luminal domain, and subsequently used this as a molecular replacement model for the mouse PERK luminal domain. The crystal structures reveal human PERK captured in a novel tetramer arrangement, while the mouse PERK luminal domain is visualized in a dimer state similar to Ire1 luminal domains.

**Table 1:** Data collection and refinement statistics

	*H. sapiens*			*M. musculus*
	Peak Na_2_WO_4_	Inflection Na_2_WO_4_	Remote Na_2_WO_4_	–
Data collection statistics				
Space group	P4_1_2_1_2	P4_1_2_1_2	P4_1_2_1_2	P3_1_21
Molecules in asym unit	2	2	2	1
Unit cell, a(b),c, Å	83.9, 186.2	84.1,186.5	84.2,186.9	87.6, 73.6
Resolution range, Å	75.6–3.1	76.8–3.57	76.8–4.0	52.8–3.3
Wavelength, Å	1.2148	1.2152	0.9795	0.9795
Completeness, %	99.9(99.5)	99.9(100.0)	99.9(99.8)	99.7(99.7)
I/σ(I)	32.3(4.4)	34.6(7.5)	38.5(7.2)	12.2(2.6)
*R*_merge_, %	4.8(65.7)	4.6(42.6)	4.2(36.9)	7.4(63.3)
Refinement statistics				
Protein atoms	3,282			1,268
*R*_work_	24.2			28.6
*R*_free_	29.3			30.6
Rmsd, Å	0.004			0.003
Rmsd, °	0.991			0.935
Ramachandran favored, %	91.2			84.1
Ramachandran outliers, %	1.2			2.0

### Crystal structure of human PERK luminal domain tetramer

**Figure 1:**
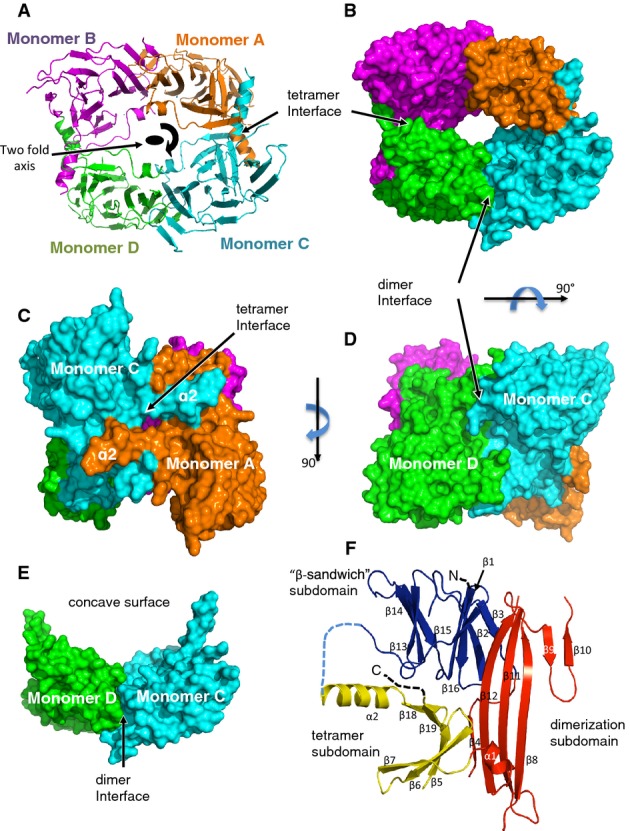
Human PERK luminal domain tetramer structure

The human PERK luminal domain structure forms a ring-type tetramer architecture. The individual monomers are arranged along a twofold rotation axis forming two sets of dimers A–B and C–D. Each dimer presents an inward-facing concave surface that intimately locks together at both ends to create a space in the center of the ring tetramer (Fig1A and B). The interaction between the dimers is offset relative to each other by 50 degrees. There are two significant interfaces between the monomers that give rise to the dimer and tetramer arrangements (Fig1C–E). The dimerization interface involves the interaction between monomers A–B and C–D. The interface is slanted by 25° compared to the twofold rotation axis through the middle of the tetramer generating a slightly skewed appearance. This is partly because the monomers within the dimer are not perfectly superimposable resulting in a small degree of asymmetry, but more so because this is an inherent characteristic of the dimer interface as observed for yeast and human Ire1 luminal domain structures. The tetramer interface involves the interaction between monomers A–C and B–D at the opposing side to the dimerization interface. There are substantial contacts between monomers within the tetramer interface, with the key feature being a helix swapped between monomers; such secondary structure swap motifs are indicative of a transient interface (Ali *et al*, 2006; Czabotar *et al*, 2013; Tan *et al*, 2014).

Cartoon representation of human PERK LD tetramer viewed from top along twofold rotation axis with monomer A in orange, monomer B in magenta, monomer C in cyan, and monomer D in green.

Top view of human PERK tetramer in molecular surface representation.

Side view of tetramer displaying the tetramer interface.

Front view of PERK tetramer showing the dimerization interface between monomers. The dimerization interface is offset from the twofold rotation axis by ˜25°.

Dimer component of PERK tetramer illustrating the concave surface as viewed from top.

Cartoon representation of an individual PERK LD monomer divided into subdomains with red representing dimerization subdomain, blue the β-sandwich subdomain, and yellow tetramer subdomain. Each secondary structural element is numbered.

The individual monomers that make up the tetramer consist predominantly of β-strands arranged into β-sheets, with two helices also present. The monomers A and C that come together to form one of the tetramer interfaces are more complete than the corresponding B and D monomers. Residues within monomers A and C are visible from 105 to 400, with the exception of a few loops connecting the secondary structural elements, which are disordered. Monomers B and D have more disorder in regions 300–320 due to the absence of crystal contacts that make up the crystal lattice (Supplementary Fig S1).

We have subdivided the luminal domain into three structural motifs related by function: dimerization subdomain, β-sandwich subdomain, and tetramer subdomain (Fig1F). The dimerization subdomain consists of a series of anti-parallel β-strands that form the dimerization interface between PERK monomers. The central feature of this subdomain is a β-sheet consisting of three long anti-parallel β-strands with β8 forming direct interactions to β8 from the opposing monomer. The “β-sandwich” subdomain consists entirely of β-strands arranged into a two-layer β-sandwich fold that is likely to stabilize the other subdomains. The tetramer subdomain consists mainly of β-strands and one α-helix that come together to create a cleft, which interacts with the opposite PERK monomer in a helix swap that most likely acts to stabilize the transient tetramer interface.

### Tetramer interface

**Figure 2:**
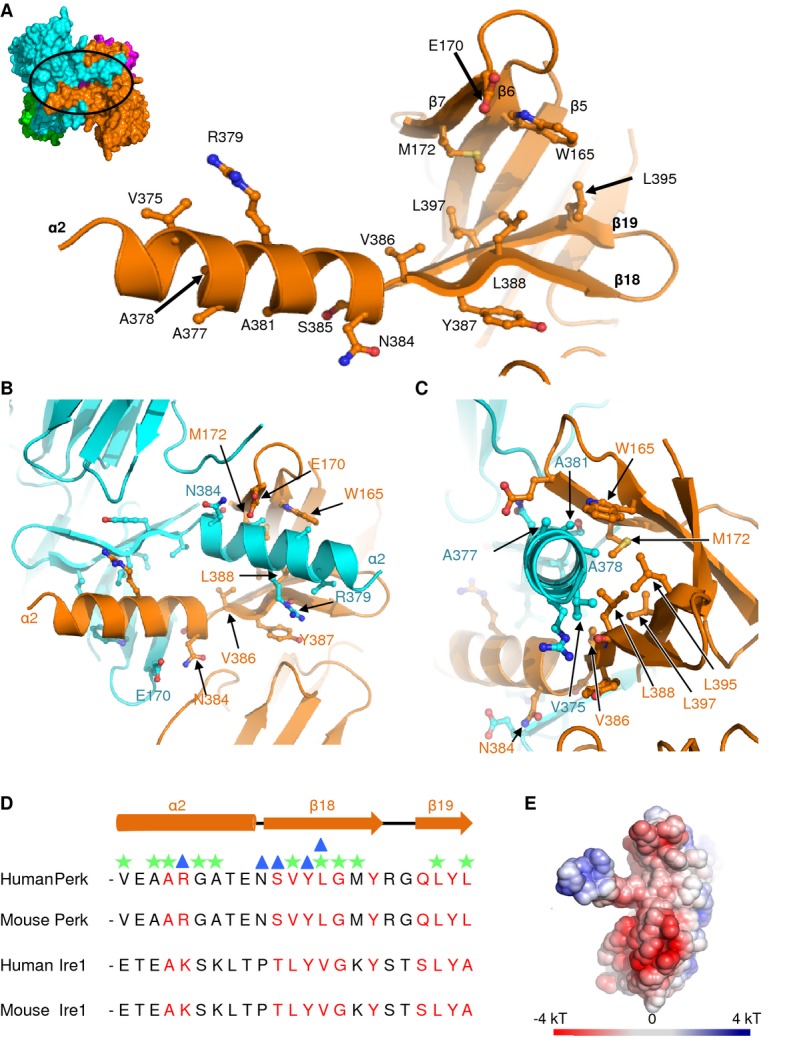
The novel tetramer interface

The salient feature of the tetramer interface is the α2 helix being swapped between opposing monomers that results in a total of 2,500 Å of solvent-accessible surface being buried, making this interface more substantial than the dimerization interface. The swapped α2 helix resides in a cleft created by β5–β7 and β18–β19 as part of the tetramer subdomain. The interface comprises predominantly hydrophobic interactions with a number of hydrogen bonds that also contribute to the interface between monomers. The most significant residues that constitute the hydrophobic core are L388, V386, and G389 from β18; L397 and L395 from β19, which together form the base of the cleft; W165 and M172 from β6 and β7, contributing from the top of the cleft (Fig2A and B). The residues V375, A377, A378, and A381 from the opposing monomer are aligned to one side of the α2 helix that faces into the cleft and constitutes the other part of the core hydrophobic interaction (Fig2C). Significant hydrogen bond interactions involve the following: N384 and S385, positioned within the base of swapped α2 helix; Y387 and L388, which are found within the β18 forming the bottom of the cleft from one monomer; E170 and M172 from the top of the cleft; and R379 from the swapped helix, respectively. Analysis of sequence alignment between Ire1 and PERK revealed that both the hydrophobic core and hydrogen bonded interactions are conserved, particularly residues located in the two β-strands β18 and β19 that form the base of the cleft (Fig2D). To further analyze the tetramer interface, we generated a surface electrostatic potential map of our structure. The electrostatic potential of α2 helix is positive while that of the cleft is negative. This clearly indicates a favorable electrostatic potential for interaction between α2 helix and tetramer subdomain cleft (Fig2E).

Residues from PERK monomer that are involved in the tetramer interface. The residues lining the binding cleft and helix α2 are predominantly hydrophobic.

Overview of molecular interactions between monomers, colored in cyan and orange, that are involved in tetramer interface.

View along the helix α2 shows the hydrophobic core interaction between the swapped helix and the binding cleft.

Alignment of PERK and Ire1 sequences from both human and mouse species. The red-colored letters denote functionally conserved residues, with green stars indicating residues involved in hydrophobic core interactions, while blue triangles indicate residues that contribute to the tetramer interface via hydrogen bond interactions.

Electrostatic surface potential representation of PERK monomer showing tetramer interface.

### Crystal structure of mouse PERK luminal domain dimer

**Figure 3:**
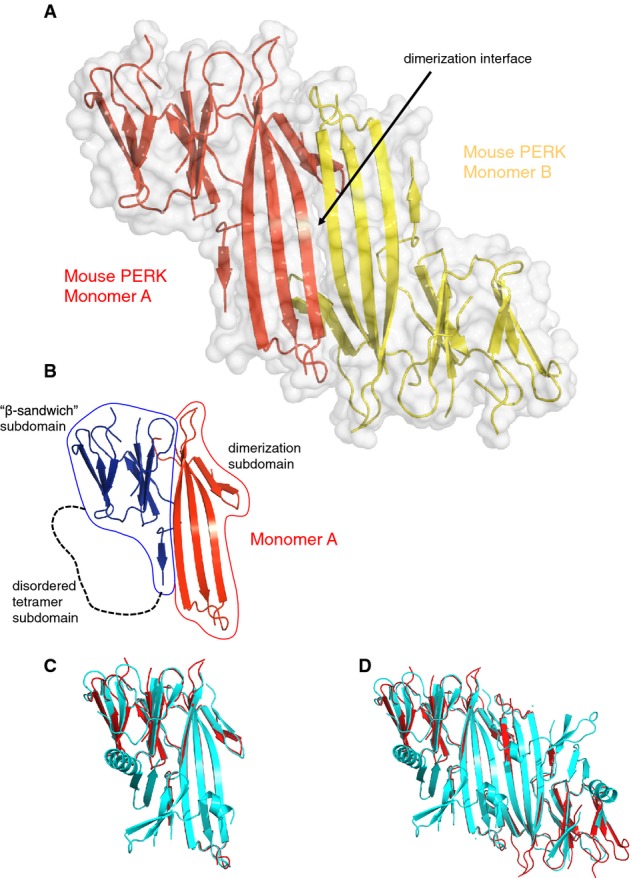
Mouse PERK luminal domain dimer structure

The crystal structure of mouse PERK luminal domain exhibits a dimer arrangement (Fig3A and B). The monomer component of the dimer structure is very similar in fold to human PERK with a route mean square deviation (rmsd) value of 1.1 Å when superimposing the two monomer structures together (Fig3C). The only significant difference between the mouse and human monomer is that in the mouse structure, the tetramer subdomain is disordered. Interestingly, while the mouse structure formed a dimer in the crystal, human PERK, which possesses an ordered tetramer subdomain, is present as a tetramer in the crystal.

Transparent molecular surface representation of dimeric mouse PERK LD crystal structure, with monomer A colored in red and monomer B in yellow.

Mouse PERK monomer organized into three subdomains with dimerization domain in red and β-sandwich domain in blue. The tetramer subdomain is disordered and is not visible within the structure.

Structural superimposition of mouse PERK (red) and human PERK (cyan) monomers. The rmsd value between the two monomers is 1.1 Å.

Structural superimposition of mouse PERK (red) and human PERK (cyan) dimers, with an rmsd value of 1.0 Å, suggests that the alignment of the dimer interface is consistent between PERK species.

Superimposition of the mouse PERK dimer with the dimer component of human PERK tetramer structure again reveals a very close match with an rmsd value of 1.0 Å (Fig3D). This indicates that the spatial arrangement of the monomers forming the dimer interface is conserved between PERK species. Furthermore, since the dimer interface is present in both dimer and tetramer structures, this suggests that this is a biologically relevant and stable dimer interface.

### Comparison of PERK and Ire1 luminal domain structures

We conducted a search for protein folds that display a homologous structural architecture to PERK in order to gain insights into function using the DALI server (Holm & Rosenstrom, 2010). As expected, we found structural similarities to luminal domains of yeast and human Ire1 only. This suggests that PERK and Ire1 luminal domains form a distinct structural class of proteins likely to have their own function unrelated to other structures within the protein database (PDB).

The PERK luminal domain structure displays a similar fold to that of yeast and human Ire1 luminal domains. Structural superposition of human PERK monomer with yeast and human Ire1 indicates rmsd values of 3.8 Å and 4.2 Å. The most significant differences between PERK and Ire1 luminal domains occur at the dimerization interface and swapped α2 helix within the PERK tetramer subdomain.

**Figure 4:**
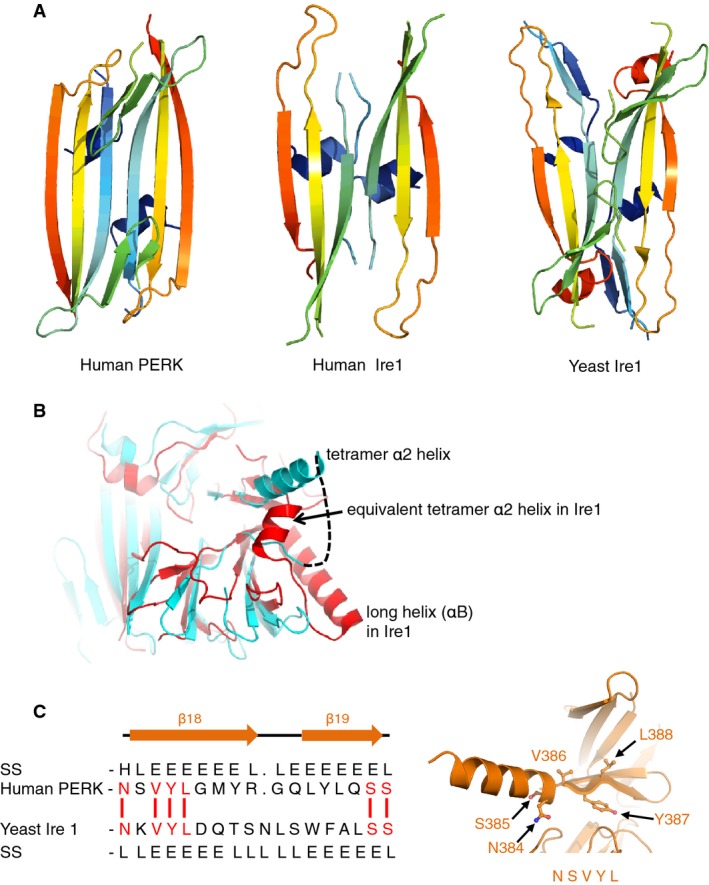
Comparison of PERK and Ire1 luminal domains structures

PERK dimerization interface is more substantial than that of human Ire1. It involves a greater number of interactions along β8 with a solvent accessible area of 2,328 Å being buried in PERK, whereas 1,732 Å is buried in human Ire1 interface. In both yeast and human Ire1 structures, the interaction of β8 is less pronounced due to high angle of alignment of monomers resulting in more curved appearance within the dimerization subdomain (Fig4A). This is compensated for in yeast by binding interactions involving β8 with α1 helix from the opposing monomer resulting in a solvent accessible area of 2,586 Å that is buried in the interface. Similarly, in human PERK, the α1 helix also contributes to the dimerization interface by interacting with β8 from the opposing monomer.

Secondary structure comparison of dimerization subdomain interface between PERK and Ire1. PERK dimer interface is greater in area compared to Ire1 due to better alignment of monomers.

Structural superimposition of human PERK (cyan) with human Ire1 (red) crystal structures. The α2 helix in PERK structure is projected outwards to form the helix-swapped tetramer interface. The equivalent helix in Ire1 is shorter and projected downward; this orientation is not conducive for helix swap to occur between monomers. The distinctively long helix (αB) observed in Ire1 structure is not present within the PERK structures—a point that is further supported by sequence alignments showing PERK lacking the long helix (αB) region (Supplementary Fig S2), and is not involved in tetramer formation.

A section from a structural pairwise alignment between human PERK and yeast Ire1 crystal structures (Supplementary Fig S3) reveals that the only significant stretch of identity (NSVYL-motif) occurs on β18, which forms the base of the cleft within the tetramer subdomain.

The PERK-swapped α2 helix is significantly different to that in Ire1 (Fig4B). The corresponding helix in human Ire1 is shorter and is orientated away from the opposing monomer. The position of α2 helix in Ire1 crystal structure is not conducive for helix swap arrangement between monomers that leads to tetramer formation. The α2 helix is preceded by a distinctively long (αB) helix in Ire1. Within PERK structure, the equivalent long helix segment is disordered and analysis of sequence indicates a low homology between PERK and Ire1 within this region; thus, it is unlikely to form a long helix in PERK. This long (αB) helix is also unlikely to be involved in tetramer formation, and hence, it is not conserved between Ire1 and PERK luminal domains (Supplementary Fig S2). In the yeast Ire1 structure, the α2 helix is disordered, similar to mouse PERK structure.

While sequence alignment between human and mouse species of PERK and Ire1 was reliable, we were less confident with yeast Ire1, particularly the C-terminal half of the luminal domain sequence. To identify regions of high similarity between human PERK and yeast Ire1, we conducted a structural pairwise alignment. We found that structural identity within the N-terminal half of the domain was similar to that predicted by sequence alignment, but the C-terminal half was different and revealed a conserved patch (NKVYL yeast Ire1, human PERK NSVYL) that represented the most significant area of structural identity between yeast Ire1 and human PERK (Fig4C and Supplementary Fig S3). This patch mapped onto β18 within the tetramer subdomain of human PERK and is intimately involved in tetramer interactions. Sequence alignment with human Ire1 had previously identified this region to have a high conservation between species, but was only obvious in yeast Ire1 sequence after structural alignment. Thus, identification of this patch and its position within the tetramer subdomain suggests that tetramer formation and any functional consequence of this event are conserved from yeast Ire1 to human PERK.

### Small angle X-ray scattering analysis of PERK luminal domain

**Figure 5:**
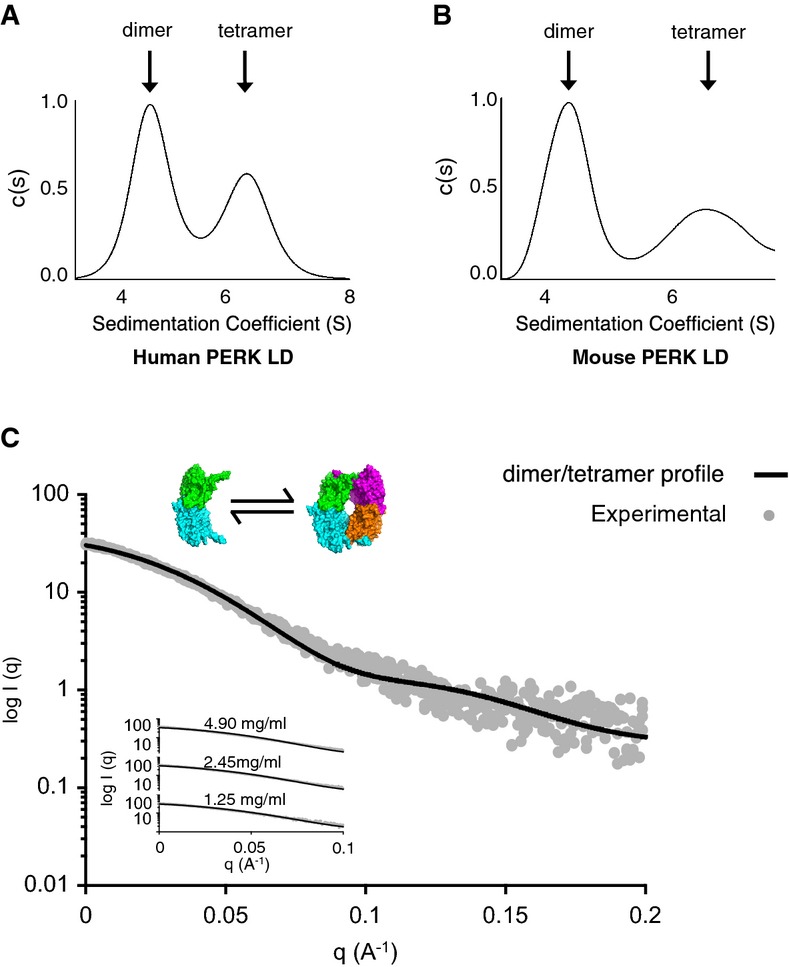
Small angle X-ray scattering (SAXS) analysis of PERK luminal domain in solution

To understand the biological relevance of the human PERK tetramer, we analyzed the oligomeric state of PERK luminal domain in solution. Firstly, we observed that human PERK luminal domain protein eluted from size exclusion chromatography–multi-angle light scattering (SEC–MALS) exclusively as a dimer (Supplementary Fig S4). However, analysis of human PERK luminal domain protein by analytical ultra centrifugation (AUC) revealed a significant tetramer species that exists in equilibrium with dimer in solution, with a dimer to tetramer ratio of 3:2 (Fig5A). To test whether mouse PERK luminal domain also forms tetramer species, we repeated AUC with mouse PERK luminal domain. We found that mouse PERK luminal domain also forms a dimer–tetramer species that exists in a similar ratio (_dim_3:2_tet_) to that of human PERK luminal domain protein (Fig5B). We did not observe any oligomer species higher than that of a tetramer. This indicates that both human and mouse PERK luminal domains form stable dimers, while the formation of tetramer occurs transiently for both proteins. Furthermore, it suggests that the association and dissociation of stable dimers to form transient tetramers may play a regulatory role in UPR signaling. The ability to crystalize the proteins in different states are purely a result of the crystallization conditions favoring that particular state, and by chance, we were able to capture both states in our crystallization experiments. Next, to confirm that the tetramer arrangement that we visualized within the crystal lattice is present in the same arrangement in solution, we preformed small angle X-ray scattering (SAXS) at concentrations between 1 and 5 mg/ml. We calculated a SAXS profile based on either our human PERK crystal structure tetramer only, our PERK dimer structure only, or a mixture of the two and then compared this to the experimentally derived SAXS solution data profile (Fig5C). We see a poor fit between calculated and experimental SAXS profiles with both a dimer (*χ* = 1.7) and tetramer (*χ* = 1.5) models only. However, when we use a mixture of dimer and tetramer in a ratio of 3:2, as suggested by AUC and reinforced by the SAXS program OLIGOMER (Konarev *et al*, 2003), we observe an excellent fit between calculated and experimental SAXS profiles (*χ* = 1.1). This clearly indicates that both the dimer and tetramer arrangements that we see within the PERK crystal structures exist in solution. Moreover, the dimer and tetramer species are in equilibrium similar to that observed by AUC. Thus, we have here captured by X-ray crystallography two biologically relevant states of PERK luminal domain that exists in solution.

Sedimentation velocity AUC profile reveals human PERK LD exists as a dimer–tetramer species in solution, in a _dimer_3:2_tetramer_ ratio, indicating the transient nature of the tetramer species, while reinforcing the stable nature of the dimer interface.

AUC analysis of mouse PERK LD also indicates that mouse PERK LD forms dimer–tetramer species in a similar ratio (_dimer_3:2_tetramer_) to that of human PERK LD.

Small angle X-ray scattering analysis of human PERK LD in solution comparing the experimental SAXS profile (gray dots) to the computed profile of PERK LD crystal structures, when using a _dimer_3:2_tetramer_ ratio of dimer–tetramer, based on AUC and further reinforced by the program OLIGOMER, resulting in an excellent fit (*χ* = 1.1). Inset, profiles for independent SAXS runs at various concentrations.

### Structure-guided mutational analysis of PERK tetramer *in vitro*

To further interrogate the biological relevance of the dimer–tetramer states, we introduced mutations into the interface that would affect tetramer formation by specifically targeting hydrophobic interactions. The mutations were as follows: W165A, situated at the top of the hydrophobic cleft; L388N, which forms part of the NSVYL-conserved tetramer patch and is positioned at the bottom of the cleft upon β18; the residues L395N and L397N, located upon β19; and A378N, the conserved residue positioned on the α2 helix, which faces into the hydrophobic core. Mutation of the Leu to Asn replaces a hydrophobic residue with that of a polar, hydrophilic residue of similar size, while mutation of Trp to Ala reduces the hydrophobicity of the residue. Thus, the mutations target the hydrophobic character of the tetramer interface.

**Figure 6:**
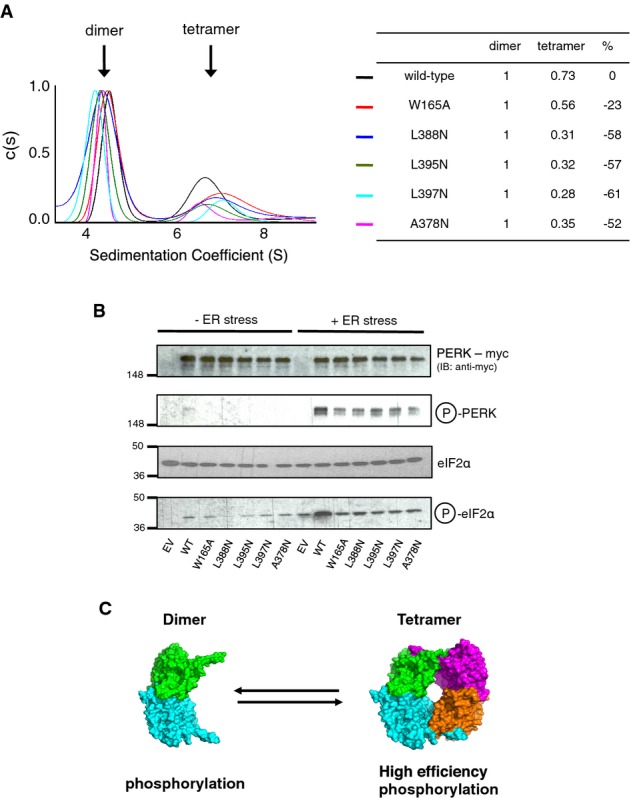
Structure-guided mutational analysis *in vitro* and *in vivo*

We employed the use of AUC to measure tetramer formation between wild-type and mutant proteins in solution (Fig6A). We found that all mutations tested reduced the percentage of tetramer observed in solution when compared to wild-type luminal domain PERK. Mutations positioned at the base of the cleft and on the α2 helix exhibited the greatest effect, reducing the amount of tetramer observed by 52–61% (Fig6A). Thus, mutations targeting the hydrophobic nature of the tetramer interface reduce PERK luminal domain tetramer formation by shifting the equilibrium in favor of dimer species in solution.

Sedimentation velocity AUC analysis comparing the levels of dimer and tetramer in solution between wild-type PERK luminal domain (black) and tetramer interface mutants: W165A (red), L388N (blue), L395N (green), L397N (cyan), and A378N (magenta). All mutations reduce tetramer formation *in vitro*, with mutations situated at the base of the hydrophobic cleft (L388N, L395N, L397N) and on the helix α2 (A378N) having the greatest effect.

PERK^−/−^ MEF cells were transfected with empty vector (EV), myc-tagged wild-type PERK (WT), and myc-tagged PERK tetramer mutants, and were assessed for PERK and eIF2α phosphorylation both in the absence and presence of 5 μm tunicamycin for 4 h to induce ER stress. After immunoblotting, we observed a reduction in the levels of PERK and eIF2α phosphorylation for mutants when compared to wild-type PERK that mirrors the effects seen *in vitro*.

Model illustrating the transition from dimer to tetramer being a likely regulatory step in UPR signal activation. Tetramer formation results in a higher efficiency of auto-phosphorylation of the PERK kinase domain.

Source data are available online for this figure.

### Impact of tetramer mutations on PERK stress signaling *in vivo*

To investigate whether tetramer formation is important for PERK signaling *in vivo*, we transfected PERK^−/−^ cells with empty vector, wild-type PERK, and PERK tetramer mutants: L388N, W165A, L395N, L397N, and A378N and assessed the levels of PERK and eIF2α phosphorylation in the absence and presence of ER stress (Fig6B). In unstressed cells, there was virtually no PERK phosphorylation observed, as expected. Upon addition of 5 μm tunicamycin to induce ER stress, we observed significant levels of PERK phosphorylation for cells transfected with wild-type PERK, but reduced levels of phosphorylation for all tetramer mutants in comparison. Similarly, we observed negligible levels of phosphorylated eIF2α in unstressed cells; however, we see a clear difference in levels of eIF2α phosphorylation between wild-type and PERK tetramer mutants, with the mutants displaying a reduced level of eIF2α phosphorylation upon addition of ER stress (Fig6B). These results mirror the effects observed for tetramer mutants for the *in vitro* analysis experiments. Therefore, these results suggest that luminal domain tetramer formation and specifically the hydrophobic nature of the tetramer interface are important to achieve high efficiency PERK and eIF2α phosphorylation in cells.

## Discussion

In this study, we shed new light on the mechanism of UPR activation by presenting crystal structures of PERK luminal domains captured in two different states. The first dimeric state has been previously described with Ire1 and suggests that both Ire1 and PERK form stable dimers. The second state is a novel tetramer arrangement of PERK luminal domain. The tetramer interface is dominated by a helix swapped between dimers that is indicative of a transient interface. This is further supported by AUC analysis that indicates both human and mouse luminal domain proteins can form dimers and tetramers in 3:2 ratio. Moreover, SAXS analysis clearly indicates the arrangements seen in the crystal lattice exist as dimer and tetramer in solution. The transient nature of the tetramer interface suggests a regulatory role in UPR activation, a notion that is supported by data showing tetramer interface mutants causing a reduction in the levels of PERK and eIF2α phosphorylation in cells. The tetramer seems to increase the efficiency of PERK auto-phosphorylation in cells and has been visualized before (Liu *et al*, 2002); while it is well established that a dimer is sufficient for auto-phosphorylation to occur in *trans*, for both PERK (Ma *et al*, 2002) and Ire1 (Shamu & Walter, 1996; Welihinda & Kaufman, 1996; Liu *et al*, 2000; Ali *et al*, 2011; Prischi *et al*, 2014), the tetramer may provide a more sturdy platform for the phosphorylation reaction to take place, thus increasing the efficiency of phosphorylation (Fig6C). Furthermore, we do not observe any differences in the affinity of interaction between tetramer mutants and BiP when compared to wild-type (Supplementary Fig S5), again suggesting that tetramer increases efficiency via positioning of cytoplasmic domains for phosphorylation and probably not by any other mode of activation. Since the luminal domain is the effector domain that dictates the oligomerization status for both Ire1 and PERK, it is easy to reconcile that the dimer and tetramer may represent lower and higher activated states, and shifting between these states maybe a key part of the UPR sensors ability to activate and respond to the accumulation of misfolded proteins within the ER. We recently described an allosteric UPR induction model that involves the dissociation of a noncanonical interaction between BiP and UPR transducer proteins, by unfolded protein binding to the canonical substrate-binding domain of BiP (Carrara *et al*, 2015), which relieves the BiP-luminal domain association, allowing the luminal domains to possibly form higher oligomeric states. Here, we show that the tetramer maybe one such activated state—possibly in addition to larger oligomeric species or clusters that have been previously reported (Kimata *et al*, 2007; Korennykh *et al*, 2008).

Interestingly, structural alignment programs strongly suggest that both Ire1 and PERK luminal domains are structurally related to each other and do not share high similarities with other structures deposited in the PDB, including MHC type proteins, and therefore likely represent a unique structural group with similar biological functions—in line with our previous observation that unfolded protein C_H_1 does not directly interact with both Ire1 and PERK luminal domain proteins (Carrara *et al*, 2015).

Taken together, the present study sheds new light on the mechanism of UPR activation by describing two different states of PERK luminal domain captured by X-ray crystallography and rationalizes differences between luminal domain structures. These data provide valuable mechanistic insights that may open the possibility for new therapeutic interventions targeting UPR in diseased states.

## Materials and Methods

### Protein expression and purification

*Homo sapiens* PERK (residues 105–403) and *Mus musculus* PERK (residues 101–399) genes were inserted into a modified version of the pET-17b vector that contains a His6 tag followed by a PreScission Protease cleavage site. PERK LD mutants were generated by site-directed mutagenesis. PERK LD WT and mutant proteins were expressed overnight at 22°C in Rosetta2 (DE3) *Escherica coli* cells (Merck). Cell pellets were lysed by sonication in 50 mM HEPES (pH 7.8), 400 mM NaCl, 10% glycerol buffer supplemented with 25 μg/ml DNase (Sigma-Aldrich), and Complete EDTA-free Protease Inhibitor tablets (Roche). Lysed cells were centrifuged at 40,000 g for 1 h, and the soluble fraction containing PERK LD was further purified by Co^2+^ affinity using pre-packed 5 ml HiTrap TALON crude columns (GE Healthcare). PERK LD was eluted with 250 mM imidazole. 10 U of PreScission Protease was added per 1 mg of purified protein, and samples were dialyzed against 2 L of 50 mM HEPES (pH 7.8) and 10% glycerol overnight at 4°C. Samples were passed through a second TALON column to remove any residual tagged proteins. Hereon, all buffers were supplemented with 2 mM TCEP. PERK LD proteins were further purified by anion-exchange chromatography using a 5-ml HiTrap Q HP column (GE Healthcare) and by size exclusion chromatography (SEC) on a HiLoad 16/60 Superdex 200 column (GE Healthcare) equilibrated with 50 mM HEPES (pH 7.8), 400 mM NaCl, 10% glycerol, and 2 mM TCEP.

### Crystallization and heavy atom derivatization

Initial *H. sapiens* PERK LD crystals were grown in hanging drops by mixing 1 μl of untagged protein (5 mg/ml) plus 1 μl of crystallization solution, containing 0.1 M Tris (pH 8.5), 0.2 M MgCl_2_, 25% w/v PEG3350, and 7% glycerol. Drops were equilibrated over 700 μl of crystallization solution at 18°C. Small bipyrimidal crystals appeared overnight. 10 rounds of re-iterative microseeding, in identical crystallization conditions, were carried out to improve *H. sapiens* PERK LD crystals. Cryoprotection was achieved by serial transfer of the cover slip holding the crystallized drop over reservoirs containing the crystallization solution with increasing concentrations of PEG3350. PEG3350 concentration was increased stepwise (by 2% w/v and 8–12 h incubation at each step) up to a final 40% w/v PEG3500 concentration. For phasing, *H. sapiens* PERK LD crystals were soaked with 2 mM Na_2_WO_4_ for 5 h and immediately flash-frozen without backsoaking. *M. musculus* PERK LD crystals were grown in hanging drops by mixing 2 μl of untagged protein (20 mg/ml) plus 1 μl of crystallization solution, containing 0.1 M MES/imidazole (pH 6.5), 0.09 sodium phosphate salts (NPS) mix (containing 0.03 M of each NaNO_3_, Na_2_HPO_4_, (NH_4_)_2_SO_4_), 12.5% w/v PEG1000, 12.5% w/v PEG3350, and 20% v/v MPD. Drops were equilibrated over 700 μl of crystallization solution at 18°C.

### Data collection and structure determination

X-ray diffraction datasets were collected at Diamond Light Source (Didcot, UK) on I-02 beamline. A three-wavelength MAD dataset was collected on heavy atoms derivatives. All diffraction images were integrated using iMosflm and then merged and scaled using Scala (CCP4). Phasing of *H. sapiens* PERK LD data was carried out using Shelx C/D/E via the AutoSharp pipeline. An initial *H. sapiens* PERK LD model was manually built by threading PolyAla chain through fragments of continuous electron density using Coot. Structure refinement was carried out using Phenix Refine and Feature Enhanced Maps (FEM) (Phenix). Model building was carried out manually using Coot. The structure of *M. musculus* PERK LD was solved by molecular replacement using the refined *H. sapiens* PERK LD structure as the search ensemble (Phaser).

### Analytical ultracentrifugation

Sedimentation velocity analytical ultracentrifugation was performed by Dr. Katherine Stott (Biochemistry Department, University of Cambridge). Experiments were carried out using a Beckman Optima XL-I centrifuge. Data were obtained over 7 h (263.2” for each time point) of centrifugation at 20°C using refractive index detection. PERK LD proteins at precisely 30 μM were analyzed by AUC in 50 mM HEPES (pH 7.8), 200 mM NaCl, and 2 mM TCEP buffer. The raw data were analyzed by Sedfit and transformed into a c(s) plot.

### SAXS

*H. sapiens* PERK LD (1.25 mg/ml) in 50 mM HEPES (pH 7.8), 200 mM NaCl, and 2 mM TCEP buffer was analyzed by SAXS. SAXS data were collected at the PETRA III P12 beamline at the DESY synchrotron (Hamburg, DE), with the assistance of Dr. Petr Konarev (EMBL c/o DESY, Hamburg). The standard beamline setup in SEC mode with a Pilatus 2M detector, set at a distance of 3.1 m, was used. Data were processed with PRIMUS (Konarev *et al*, 2003) by Dr. Petr Konarev (EMBL c/o DESY, Hamburg). Different oligomer assemblies of the X-ray crystal structure *H. sapiens* PERK LD (dimers and tetramers) were used as models to fit the SAXS data. OLIGOMER program was used to determine the ratio of dimer to tetramer assemblies that best fit the experimental data, which was in agreement with AUC. The quality of the fit was assessed using chi-values output by OLIGOMER.

### Sec-mals

For SEC-MALS experiments, an Agilent 1260 (Agilent Technologies) system equipped with a miniDAWN TREOS (Wyatt Technologies) light scattering (LS) detector and an Optilab T-rEX (Wyatt Technologies) refractive index (RI) detector was used. A Superdex 200 PC 3.2/30 column (GE Healthcare) was pre-equilibrated with in 50 mM HEPES (pH 7.8), 200 mM NaCl, and 2 mM TCEP buffer until LS and RI readings were stable. A total of 200 μl of *H. sapiens* PERK LD proteins at 100 μM was injected, and LS and RI values were recorded. Peaks of interest were manually selected, and the data were analyzed using the ASTRA software (Wyatt Technologies) to calculate MW values and the polydispersity of the sample.

### Cell culture

PERK^−/−^ MEF cells were cultured in Dulbecco's modified Eagle's medium supplemented with 10% fetal bovine serum, 2 mM l-glutamine, and 50 U penicillin/50 μg streptomycin/ml. A day before transfection, 300,000 cells/well (2 ml) were plated on 6-well plate. Wells were transfected with 2.5 μg of DNA (empty vector/wild-type/mutant PERKcloned into pcDNA3 vector) and mixed with Fugene HD reagent (Promega) in ratio 1:6. 48 h after transfection, cells were either harvested (unstressed samples) or induced with 5 μM tunicamycin dissolved in DMSO (0.5% v/v), and harvested after 4 h (ER stressed samples).

### Immunoblotting

Cell monolayers in wells were washed two times in ice-cold PBS and lysed in HEPES–Triton X-100 buffer supplemented with protease/phosphatase inhibitors (20 mM HEPES, pH 7.5, 150 mM NaCl, 1% Triton X-100, 10% glycerol, 1 mM EDTA, 10 mM sodium diphosphate, 100 mM NaF, 17.5 mM B-glycerophosphate, 1 mM phenylmethylsulphonyl fluoride, 4 μg/ml aprotonin, and 2 μg/ml pepstatin A). Next, cells were scraped at 4°C and incubated on ice for 5 min. Lysates were then cleared by centrifugation at 16,800 *g* for 10 min at 4°C. Supernatant samples were mixed with Leammli buffer and run on 3–8% precast Tris-Acetate gel (Invitrogen). Gels were transferred to nitrocellulose membrane (Invitrogen‘s iBlot) and blocked in TBST + 5% non-fat dry milk. Primary antibody was added to blocking buffer in concentration of 1:1,000 for anti-c-Myc (Abcam)-tagged PERK for total PERK levels, 1:200 for phospho-specific anti-PERK (Thr981) (Santa Cruz Biotechnology), 1:500 for phospho-specific anti-eIF2α (S51) (Abcam), and 1:1,000 for total eIF2α levels (Cell Signaling). After overnight incubation at 4°C, membranes were washed three times in TBST buffer and incubated with either anti-rabbit-HRP 1:4,000 (Cell Signaling Technology) or anti-mouse-HRP antibody 1:6,000 (GE Healthcare). Secondary antibody was added to membranes in 5% milk-TBST, left at 4°C for one hour, and then washed three times. Blots were visualized by Millipore Luminata Crescendo Western HRP substrate and developed on Amersham Hyperfilm ECL (GE Healthcare).

### Accession numbers

Structure coordinates, 4YZS and 4YZY, relating to human PERK and mouse PERK structures have been deposited to the PDB.

## Supporting Information

Supplementary Figures S1–S5

Review Process File

## References

[b1] Ali MMU, Roe SM, Vaughan CK, Meyer P, Panaretou B, Piper PW, Prodromou C, Pearl LH (2006). Crystal structure of an Hsp90–nucleotide–p23/Sba1 closed chaperone complex. Nature Publishing Group.

[b2] Ali MMU, Bagratuni T, Davenport EL, Nowak PR, Silva-Santisteban MC, Hardcastle A, McAndrews C, Rowlands MG, Morgan GJ, Aherne W, Collins I, Davies FE, Pearl LH (2011). Structure of the Ire1 autophosphorylation complex and implications for the unfolded protein response. The EMBO journal.

[b3] Bertolotti A, Zhang Y, Hendershot LM, Harding HP, Ron D (2000). Dynamic interaction of BiP and ER stress transducers in the unfolded-protein response. Nat Cell Biol.

[b4] Carrara M, Prischi F, Ali MMU (2013). UPR signal activation by luminal sensor domains. Int J Mol Sci.

[b5] Carrara M, Prischi F, Nowak PR, Kopp MC, Ali MMU (2015). Noncanonical binding of BiP ATPase domain to Ire1 and Perk is dissociated by unfolded protein CH1 to initiate ER stress signaling. eLife.

[b6] Credle JJ, Finer-Moore JS, Papa FR, Stroud RM, Walter P (2005). On the mechanism of sensing unfolded protein in the endoplasmic reticulum. Proc Natl Acad Sci USA.

[b7] Czabotar PE, Westphal D, Dewson G, Ma S, Hockings C, Fairlie WD, Lee EF, Yao S, Robin AY, Smith BJ, Huang DCS, Kluck RM, Adams JM, Colman PM (2013). Bax crystal structures reveal how BH3 domains activate Bax and nucleate its oligomerization to induce apoptosis. Cell.

[b8] Gardner BM, Walter P (2011). Unfolded proteins are Ire1-activating ligands that directly induce the unfolded protein response. Science.

[b9] Hetz C, Martinon F, Rodriguez D, Glimcher LH (2011). The unfolded protein response: integrating stress signals through the stress sensor IRE1{alpha}. Physiol Rev.

[b10] Holm L, Rosenstrom P (2010). Dali server: conservation mapping in 3D. Nucleic Acids Res.

[b11] Kimata Y, Ishiwata-Kimata Y, Ito T, Hirata A, Suzuki T, Oikawa D, Takeuchi M, Kohno K (2007). Two regulatory steps of ER-stress sensor Ire1 involving its cluster formation and interaction with unfolded proteins. J Cell Biol.

[b12] Konarev PV, Volkov VV, Sokolova AV, Koch MHJ, Svergun DI (2003). PRIMUS: a Windows PC-based system for small-angle scattering data analysis. J Appl Crystallogr.

[b13] Korennykh AV, Egea PF, Korostelev AA, Finer-Moore J, Zhang C, Shokat KM, Stroud RM, Walter P (2008). The unfolded protein response signals through high-order assembly of Ire1. Nature.

[b14] Korennykh A, Walter P (2012). Structural basis of the unfolded protein response. Annu Rev Cell Dev Biol.

[b15] Liu CY, Schroder M, Kaufman RJ (2000). Ligand-independent dimerization activates the stress response kinases IRE1 and PERK in the lumen of the endoplasmic reticulum. J Biol Chem.

[b16] Liu CY, Wong HN, Schauerte JA, Kaufman RJ (2002). The protein kinase/endoribonuclease IRE1alpha that signals the unfolded protein response has a luminal N-terminal ligand-independent dimerization domain. J Biol Chem.

[b17] Ma K, Vattem KM, Wek RC (2002). Dimerization and release of molecular chaperone inhibition facilitate activation of eukaryotic initiation factor-2 kinase in response to endoplasmic reticulum stress. J Biol Chem.

[b18] Parmar VM, Schröder M (2012). Sensing endoplasmic reticulum stress. Adv Exp Med Biol.

[b19] Prischi F, Nowak PR, Carrara M, Ali MMU (2014). Phosphoregulation of Ire1 RNase splicing activity. Nat Commun.

[b20] Ron D, Walter P (2007). Signal integration in the endoplasmic reticulum unfolded protein response. Nat Rev Mol Cell Biol.

[b21] Schröder M, Kaufman RJ (2005). The mammalian unfolded protein response. Annu Rev Biochem.

[b22] Shamu CE, Walter P (1996). Oligomerization and phosphorylation of the Ire1p kinase during intracellular signaling from the endoplasmic reticulum to the nucleus. EMBO J.

[b23] Tan K, Chhor G, Binkowski TA, Jedrzejczak RP, Makowska-Grzyska M, Joachimiak A (2014). Sensor domain of histidine kinase KinB of Pseudomonas – a helix-swapped dimer. J Biol Chem.

[b24] Walter P, Ron D (2011). The unfolded protein response: from stress pathway to homeostatic regulation. Science.

[b25] Welihinda A, Kaufman R (1996). The unfolded protein response pathway in Saccharomyces cerevisiae. J Biol Chem.

[b26] Zhang K, Kaufman RJ (2008). From endoplasmic-reticulum stress to the inflammatory response. Nature.

[b27] Zhou J, Liu CY, Back SH, Clark RL, Peisach D, Xu Z, Kaufman RJ (2006). The crystal structure of human IRE1 luminal domain reveals a conserved dimerization interface required for activation of the unfolded protein response. Proc Natl Acad Sci USA.

