# Supplementary material for: Crystal structures reveal transient PERK luminal domain tetramerization in endoplasmic reticulum stress signaling
Source: EMBO J. 2015 Apr 29;34(11):1589–600. doi: 10.15252/embj.201489183 (PMC4474532; doi:10.15252/embj.201489183)
Supplement: Supplementary file 1 [file embj0034-1589-sd1.pdf]

Supplementary Figures

Figure S1

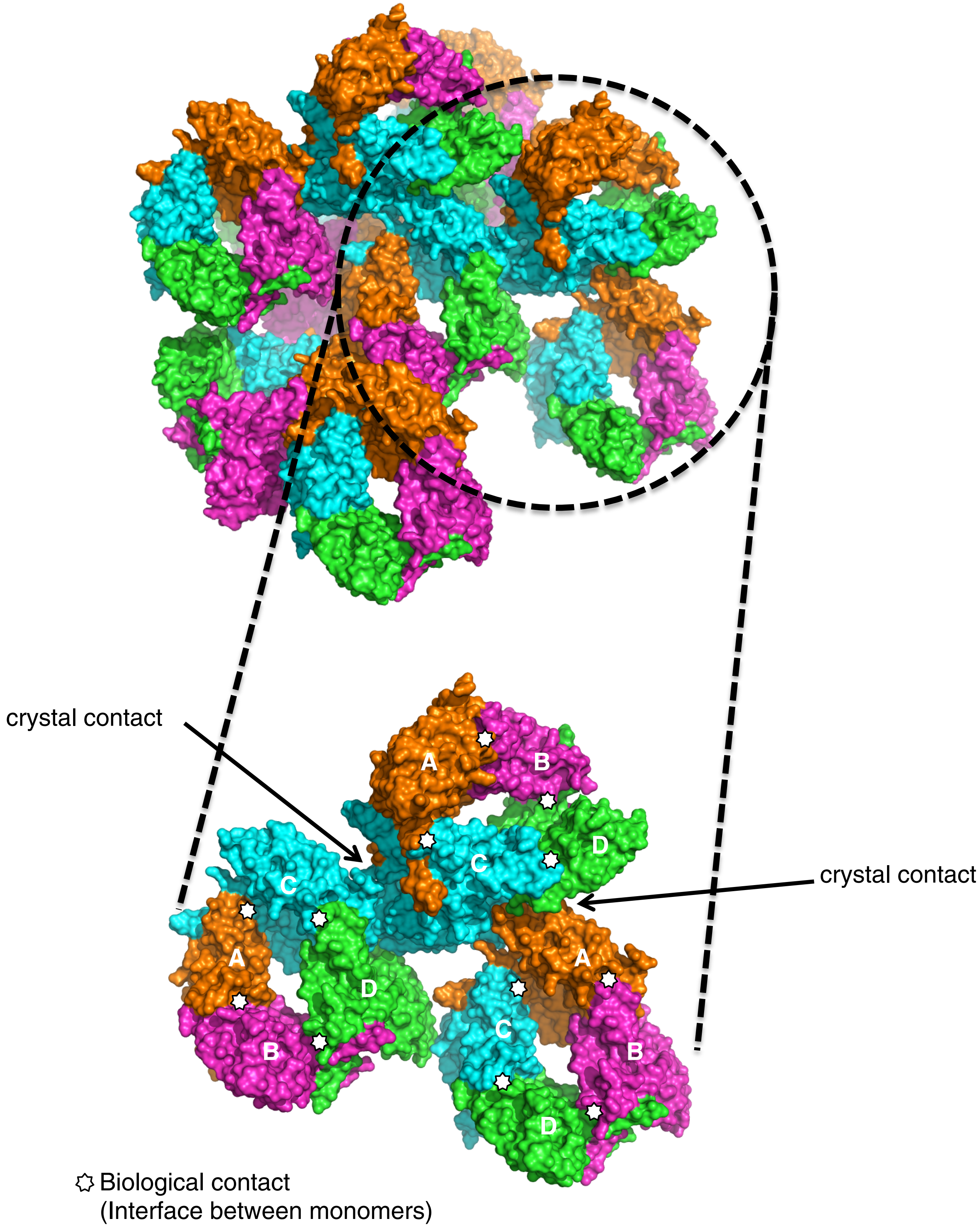

Figure S2

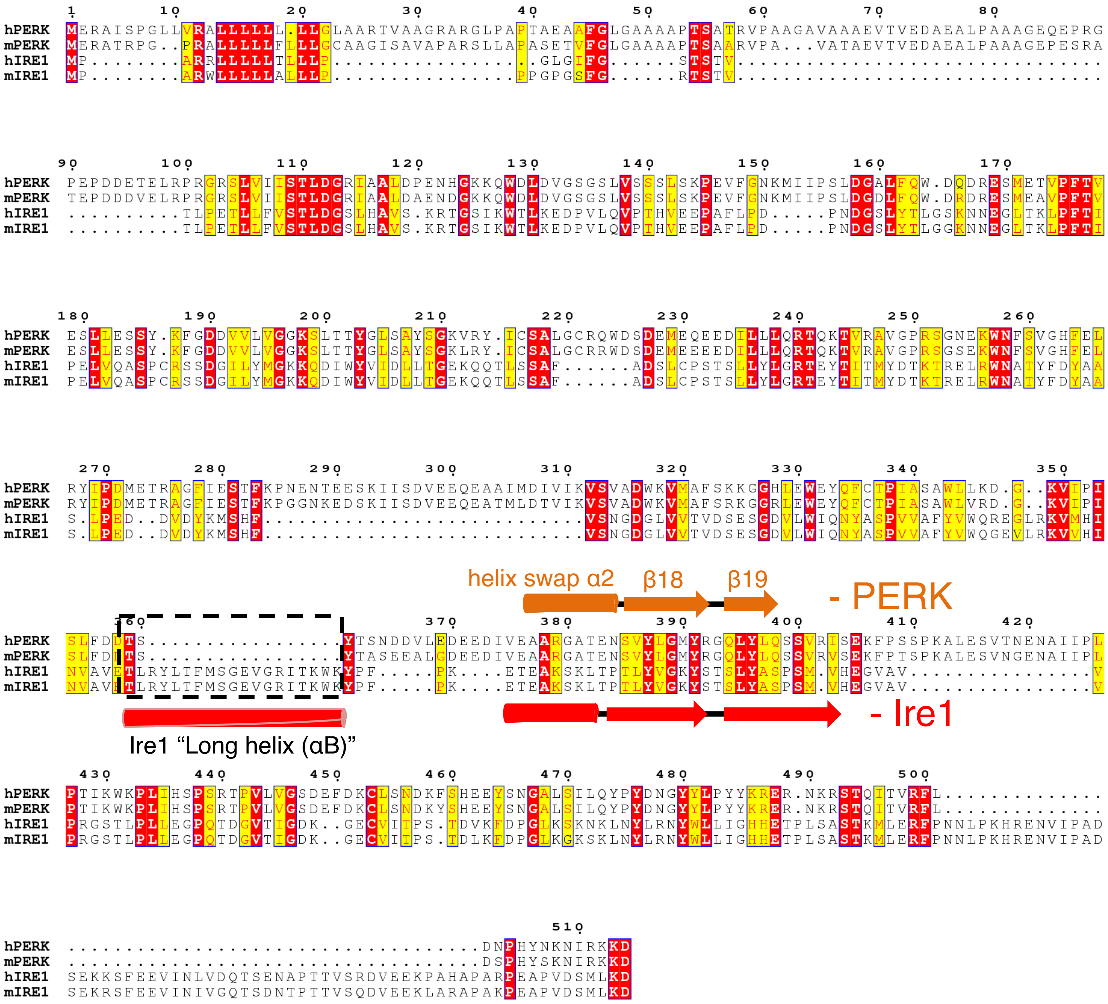
hPERK
mPERK
hIRE1
mIRE1
helix swap α2
β18
β19
- PERK
- Ire1
Ire1 “Long helix (αB)”

Figure S3

Structural pairwise alignment of yeast Ire1 with human PERK (RMSD 3.8Å)

```
DSSP  llllllleeeEEEEEEELLLLEEEEELL.LLEEEEEELHHhLLLLEELLLlllllllEEE
yIre1 nrslnelslsDILIAADVEGGLHAVDRR.NGHIIWSIEPEnFQPLIEIQEpsrletyETL   59
                   |     |   | |    |   |         |
hPerk ..........SLVIISTLDGRIAALDPEnHGKKQWDLDVG.SGSLVSSSL.......KMI   42
DSSP  ..........LLEEEEELLLLEEEEELLlLLEELLLLLLL.LLLLEEEEL.......LEE


DSSP  EELLLllLEEEEEEL.LLEEEEEEEEHHHHHLLlleeeellllLEEEEELEEEEEEEEEEL
yIre1 IIEPFgdGNIYYFNA.HQGLQKLPLSIRQLVSTsplhlktneDEKVYTGSMRTIMYTINM  118
      |      |               |     |            |  |  |      |
hPerk IPSLD..GALFQWDRdRESMETVPFTVESLLES.........DDVVLVGGKSLTTYGLSA   91
DSSP  EELLL..LEEEEEELlLLEEEEEEEEHHHHHLL.........LLLEEEEEEEEEEEEELL


DSSP  LLLLEEEEELLLLLLL.llEEEEEEEEEELEELLL.LLLL.LLLEEEEEEELLlllhhhhl
yIre1 LNGEIISAFGPGSKNG.enMIVIGKTIFELGIHSY.DGAS.YNVTYSTWQQNvldvplal  175
        |                      |           |    |
hPerk YSGKVRYICSALGCRQwdsILLLQRTQKTVRAVGPrSGNEkWNFSVGHFELR........  143
DSSP  LLLLEEEEELLLLEEElllLEEEEEEEEEEEEELLlLLLLlEEEEEEEEEEE........

DSSP  lllLLLLLLLEEEE.LLLEEEEEELLLLLEEEELlLLLLLEEEEEEEEEelllleEEEEE
yIre1 qntFSKDGMCIAPF.RDKSLLASDLDFRIARWVSpTFPGIIVGLFDVFNdlrtneNILVP  234
                |     |    |         |    |   |                  |
hPerk yipAAIMDIVIKVSvADWKVMAFSKKGGHLEWEY.QFCTPIASAWLLKD......GKVIP  196
DSSP  ellLLLLLLEEEEEElLLLEEEEEEELLLLEEEEE.ELLLLEEEEEEEEL......LEEEE


DSSP  LL................lllLLEEEEELLlLLEEEEELlllhhhhhlllllhhhhlhhh
yIre1 HP................fnpNKVYLDQTSnLSWFALSSqnfpslvesapisryassdrw  278
                           | |||           ||
hPerk ISlfddtseediveaargateNSVYLGMYR.GQLYLQSS.....................  234
DSSP  LLlllllllllhhhhhhhhhhHLEEEEEEL.LEEEEEEL.....................


DSSP  llhhhhhlhhhhhhhhleeelll
yIre1 rvssifedetlfknaimgvhqiy  301

hPerk .......................  234
DSSP  .......................
```

Figure S4

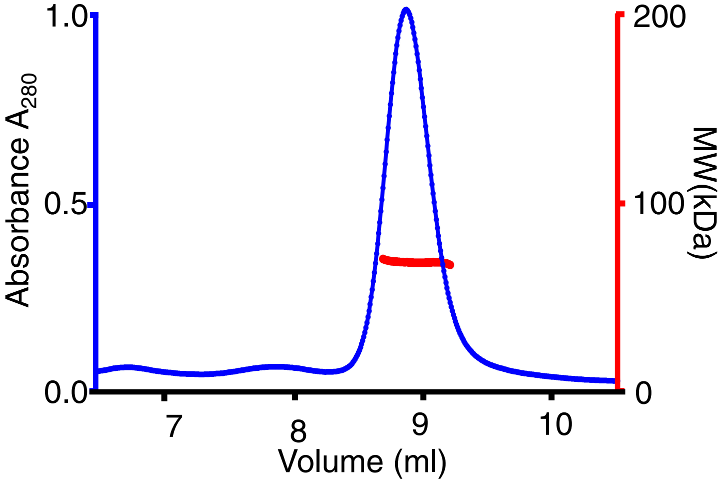

Figure S5

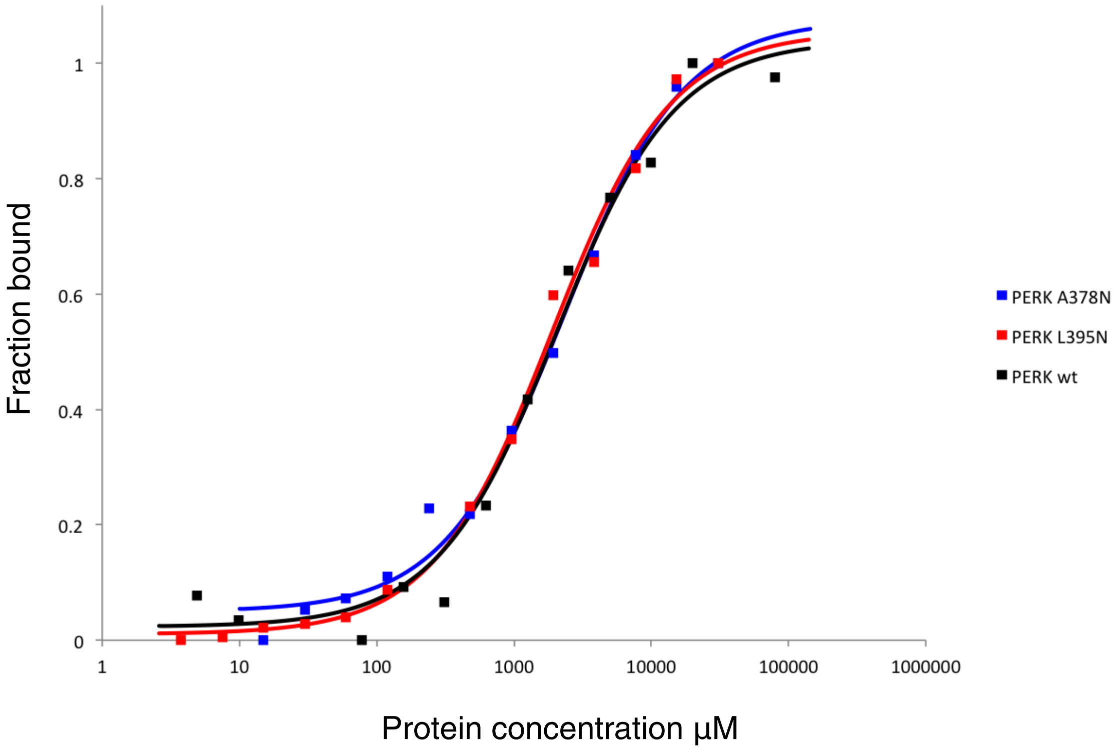
Fraction bound
1
0.8
0.6
0.4
0.2
0
1
10
100
1000
10000
100000
1000000
Protein concentration µM
PERK A378N
PERK L395N
PERK wt

Figure S1 human PERK crystal packing arrangement.
Crystals form a tetragonal arranged lattice in space group $p4_12_12$, with two molecules in asym unit. From the crystal lattice arrangement, PERK tetramers are immediately obvious and clearly suggest biological relevance. This is further supported by functional analysis including SAXS measurements. Crystal contacts that are necessary to make up the crystal lattice, and have no biological role, are also labeled.

Figure S2 Sequence alignment between PERK and Ire1 luminal domains.
Sequence alignment between human and mouse species of PERK and Ire1, highlighting the low sequence conservation for long helix (αB) in Ire1 when compared to PERK. This suggests that this particular helix (αB) present in Ire1 is not involved in tetramer formation.

Figure S3 Structural alignment between yeast Ire1 and human PERK.
Structural alignment reveals the most significant area of identity between luminal domains is located within β18 within tetramer subdomain of PERK (highlighted in red). The conserved patch (NKVYL – Ire1, NSVYL – PERK) suggests that tetramer formation and any functional consequences of this event are conserved from yeast Ire1 to human PERK.

Figure S4 SEC-MALS analysis of human PERK LD.
Human PERK luminal domain elutes as a dimer after SEC-MALS analysis.

Figure S5 Interaction analysis of wild type, L395N and A378N PERK with BiP.
Microscale thermophoresis binding curves measuring the K_d of interaction between wild type (2.0μM), L395N (1.8μM), and A378N (2.3μM) PERK with BiP.
