# Supplementary material for: Crystal structures reveal transient PERK luminal domain tetramerization in endoplasmic reticulum stress signaling
Source: EMBO J. 2015 Apr 29;34(11):1589–600. doi: 10.15252/embj.201489183 (PMC4474532; doi:10.15252/embj.201489183)
Supplement: Supplementary file 2 [file embj0034-1589-sd2.pdf]

THE
EMBO
JOURNAL

Manuscript EMBO-2014-89183

Crystal structures reveal transient PERK luminal domain tetramerization in ER stress signaling

Marta Carrara, Filippo Prischi, Piotr R Nowak, Maruf M.U. Ali

Corresponding author: Maruf M. U. Ali, Imperial College London

Review timeline:

Submission date:	04 June 2014
Editorial Decision:	18 July 2014
Re-submission:	16 February 2015
Editorial Decision:	17 March 2015
Revision received:	08 April 2015
Accepted:	13 April 2015

Editor: Alexander Kohlmaier, Hartmut Vodermaier

Transaction Report:

(Note: With the exception of the correction of typographical or spelling errors that could be a source of ambiguity, letters and reports are not edited. The original formatting of letters and referee reports may not be reflected in this compilation.)

1st Editorial Decision 18 July 2014

Thank you for submitting your study " Crystal Structures of Perk Luminal Domains Reveal Transient Tetramer Important for Stress Signaling" for consideration in the EMBO Journal. We have now received the reports of three expert reviewers, which you will find copied below. As you will see, the referees' specific comments are well-considered and constructive. I have to come to the conclusion, however, that your manuscript did not receive the neceessary strong support by the referees to warrant publication in the EMBO Journal at this point.

While at least two referees acknowledge that your findings are of interest in principle, all of them voice a number of substantial concerns and considerations that question whether the proposed dimer arrangement and transition to tetrameric arrangement of human PERK in vitro reflects a biologically relevant organisation. This pertains not only to the discrepancy between mouse and human PERK tetramerisation capacity, but, more importantly in our view, to the lack of further-reaching experiments and controls that could fully conclusively demonstrate the relevance of human tetramerisation for UPR activation in vivo. These concerns were shared and explained in detail by referees #1 and #2. Whether this process is indeed not functional in mouse remains to be tested as well. Moreover, during mutation analysis, the role of tetramerisation was not fully compellingly distinguished from effects on PERK stability, a concern shared by referees #2 and #3. Finally, also other substantial points and technical concerns remained open, which I will not repeat here in detail. I hope you appreciate that the nature and number of concerns leave me little choice but to conclude that we will not be able to offer publication of the presented work in their current form.

On the other hand, we do appreciate your approach, and in this special case, we would like to offer to you to look at your study once more, should your work in addressing the reviewers major points

allow to obtain decisive evidence in support of your model: Please re-upload afresh such a modified version of your manuscript for evaluation to our journal, and we can offer to send this manuscript to our initial referees, provided the novelty of the findings would still be uncompromised at the time of re-submission. We realize that the experimental revision requested is substantive, and we would also understand if you regard the option we are offering as beyond the scope of the current project and decide to submit your manuscript elsewhere.

****

Referee #1:

The manuscript by Carrara and colleagues solved the crystal structure of human and mouse PERK capturing two states of organization: dimers and tetramers. Interestingly, human PERK is able to form dimers and tetramers; however, mouse PERK only form dimers. In addition, these organization states of human PERK might impact PERK auto-transphosphorylation and signaling under ER stress conditions suggesting that the transition between dimers and tetramers might have a regulatory role in PERK signaling. Overall the results and crystallographic data are convincing and well presented, some additional experiments are required to address the biological role of the transition between dimers and tetramers in UPR signaling.

General comments:
The results described in this manuscript are interesting and might have a positive impact in the UPR field defining this novel dimerization state of human PERK. Some experiments describing the role of these dimerization states in PERK signaling are required

Specific comments

Regarding the mouse PERK structure, multiple alignment analysis shown in this manuscript describe that the sequences comprising the tetramer subdomain is highly conserved between mouse and human PERK (Figure 2D). As a non-expert in crystallographic and structural analysis, we ask why is the mouse PERK tetramer subdomain disordered compared with human PERK? This should be further discussed and tested. And why, this tetramer subdomain is most likely to be also present in human IRE1, if the sequence similarity is more different than mouse PERK? It is possible that this disordered tetramer subdomain in mouse PERK can be due to some issues regarding building of the structure from crystallographic data.

In the same idea, SEC-MALS and analytical ultracentrifugation was only done with the luminal domain of human PERK. Is it possible to identify dimers and tetramers in solution of luminal domain of mouse PERK?

Although the authors evaluated the formation of tetramers in solution using analytical ultracentrifugation, it will be useful to evaluate if the tretrameric conformation is also present in vivo. Some experiments using crosslinking of proteins could be done to evaluate if these tetramers are in fact present on a cellular context and if the transition from dimeric to tetrameric states of human PERK is dependent of ER stress. This is a key experiment to provide.

In addition, the authors discuss the possibility that these dimeric and tetrameric states are part of a regulatory step in PERK activation. If so, Do the WT PERK and W165A or L388N varianst have the same affinity of interaction with BiP? Can this explain the differences in activation of PERK? Co-IP experiments could be performed to address this point.

In figure 6A, the authors generate a series of point mutations based in structural analysis of the tetramer subdomain W165A and L388N partially abolish the formation of tetramers. In addition, this phenotype correlates with less activation of PERK evaluated by phospho-PERK under ER stress conditions. Although these experiments suggest decreased activation, HT1080 cells have endogenous PERK so this can mask the real impact of W165A and L388N mutants in PERK signaling. A PERK KO background could be used if possible.

In addition, a more detailed analysis of PERK signaling should be evaluates with other parameters

like eIF2 phosphorylation and ATF4 or CHOP expression.

If mouse PERK does not form tetramers, important controls can be done generating mutants of mouse PERK (W165A and L388N) and evaluate its activation. If this is the case, these mutants should not affect the activation of mouse PERK under ER stress conditions.

IRE1a has been suggested that IRE1a can assembly into oligomers to form clusters (Korennykh et al, Nature, 2009 and Li et al, PNAS, 2010). Generation of PERK oligomers in vivo should be tested.

Minor comments:
Some typographical errors are found in the text.

In figure 5C, in would be informative to show the other calculated and experimental SAXS analysis for proper comparison.

In figure 6B, total PERK blots must be shown and a quantitation of p-PERK/PERK should be provided.

Referee #2:

The authors describe the crystal structure of the luminal domain of the unfolded protein response (UPR) effector human PERK in a novel tetramer configuration. Tetramerization involves a helix swap mechanism, between dimers in a previously observed (and predicted) dimer configuration adopted by the luminal domain of human and yeast Ire1. Yeast Ire1 dimers but not human Ire1 dimers also assemble into a higher order filament arrangement but one that is totally different from the tetramer arrangement shown here for human PERK.

Based on the presented data, I have little confidence that the tetramer arrangement presented for the human PERK luminal domain is biologically significant. More supporting data is required to prove biological relevance and to warrant publication of this manuscript in the EMBO journal.

The luminal Sensor domains of the UPR effectors
Crystal Structure Dimer Tetramer/Oligomer 2 helix (Swap)
Yeast IRE1 YES YES Oligomer Disordered
Human IRE1 YES NO Present but   conformation
Mouse PERK YES NO Disordered
Human PERK YES YES Tetramer Present

Major points that need addressing:
1) What is the level of conservation of the tetramerization interface between human and mouse PERK? In particular the tetramerization sub-element helix 2. Since mouse and human PERK are highly similar proteins, it is surprising that one forms a tetramer while the other does not. This discrepancy strongly hints that the tetramer arrangement may be a species-specific artifact of high protein concentrations, which is not uncommon.

2) The tetramerization interface has not been extensively validated by mutagenesis. Only two mutants W165A and L388N were tested and both do not completely abolish tetramer formation in vitro, nor PERK autophosphorylation in vivo. Additional mutants are required to validate the tetramerization model for PERK activation. The authors should consider introducing large bulky residues or charge repulsive substitutions into helix 2 to more aggressively perturb the tetramer interaction.

3) In Figure 6 the authors seek to address whether PERK tetramerization influences PERK kinase domain activation ? Since PERK kinase domain functions as a back-to-back dimer, perhaps tetramerization is important for enabling one PERK dimer to phosphorylate another PERK dimer during the autophosphorylation reaction. The authors show a modest decrease in PERK pThr980 levels for a tetramer interface mutant but total PERK levels were also decreased so one cannot make a solid conclusion. The authors did not measure pSer51 eIF2 levels, which is the gold standard for assessing UPR activation. Stronger mutants and equal levels of protein expression are required to

make a compelling case that the tetramer configuration shown in the crystal structure is biologically relevant and not simply an artifact.

4) The authors argue that the PERK luminal domain functions as a constitutive dimer that transitions into a tetramer for activation (Figure7). Is there any evidence of a constitutive PERK dimer in vivo? Is there evidence that BiP preferentially binds luminal domain dimers vs. monomers? Does BiP binding inhibit tetramerization? More experimental data is required to support the proposed model. Currently the proposed model is highly speculative.

5) Does the oligomerization surface of yeast IRE1 overlap with the tetramerization interface of human PERK? Oligomerization of yeast IRE1 luminal domain was proposed to be induced by direct binding of unfolded proteins. Might PERK tetramerization allow for direct binding of unfolded proteins?

Minor points:
1) Alignment in Figure 2D is missing residue numbers, as referenced to human PERK.

Referee #3:

This manuscript describes a structural study of the luminal domain of the endoplasmic reticulum stress sensing protein kinase, Perk, which plays a key role in the signaling processes that accompany the unfolded protein response (UPR). The structural studies feed into a series of downstream biophysical and cellular studies that test the implications of the crystal structures, and suggest a role for tetramer formation in Perk function in the UPR. Overall the manuscript is very clearly written, the figures are effective, and for the most part the conclusions are well supported by the data presented.

The only serious shortcoming occurs in the downstream experiments where the authors engineer mutations designed to prevent tetramer formation and validate the effect of these in vitro by AUC and SAXS. They then measure consequences of these mutations on Perk phosphorylation (presumably auto-phosphorylation but not clearly stated as such) in cells following conventional UPR induction by tunicamycin. Consistent with their hypothesis that tetramer formation is functionally important the western blots show decreased signal for phosphorylated Perk at each time point for the mutants compared to the wild-type. However, the only control presented is a GAPDH loading control, which only assures that more or less equal numbers of cells were analysed in each lane.

It is essential before this data can be used, that the authors also blot for total Perk and show this alongside the phospho-Perk signal. Without this control they cannot reliably conclude that the mutations impair the mechanism of Perk activation and signalling rather than simply destabilize the protein. It would be nice, though perhaps not essential, if they also provided fluorescence microscopy data showing that the ER localization of the mutant protein is not affected by the mutations.

The authors provide a useful discussion of the mechanistic implications of their model and reasonably speculate on the basis of conservation that it might also occur in Ire1 - at least in mammalian species. It would be interesting to see some discussion of the implications of this for unraveling the complicated and somewhat controversial models (especially for Ire1) of how luminal association of these transmembrane proteins promotes association and activation of their cytoplasmic kinase domains. The senior author did provide one of the more important papers in that controversy a few years ago, so it would be good to read how they bring this new luminal tetramerisation model to bear on this issue.

Re-submission 16 February 2015

Response to reviewer's comments

We would like to thank all of the reviewers for their excellent and constructive comments; we present significant new data which shows that both mouse and human Perk LD forms dimers and tetramers in a $_{dim}3:2_{tet}$. We also now have analyzed 5 mutations both in vitro and in vivo, measuring Perk-myc as total perk levels, phosphorylated Perk and phosphorylated eIF2A levels in Perk -/- cells. We have refined the discussion to focus on our data and removed the speculative model. Together, we believe the manuscript is significantly improved!

Reviewer 1

We thank this reviewer for their positive comments!

Regarding the mouse PERK structure, multiple alignment analysis shown in this manuscript describe that the sequences comprising the tetramer subdomain is highly conserved between mouse and human PERK (Figure 2D). As a non-expert in crystallographic and structural analysis, we ask why is the mouse PERK tetramer subdomain disordered compared with human PERK? This should be further discussed and tested. And why, this tetramer subdomain is most likely to be also present in human IRE1, if the sequence similarity is more different than mouse PERK? It is possible that this disordered tetramer subdomain in mouse PERK can be due to some issues regarding building of the structure from crystallographic data.

Disorder within a crystal structure can be due to many reasons; one reason could be because that region is flexible as part of its biological function and hence is not well ordered in the electron density map for the crystallographer to build. Additionally, flexibility maybe a function of the crystal conditions itself. Also, if a structure has a crystal contact at a particular point, this point maybe better defined than at another point. We provide new evidence to show that both mouse and human LD domains form dimers and tetramers in solution Fig 5A and Fig 5B. In our tetramer structure, the tetramer subdomain is ordered since it is involved in tetramer interactions. In our dimer structure the tetramer subdomain is not involved in interactions and hence is flexible and disordered in the tetramer structure. We believe the difference in capturing these two states lies solely in the crystallization conditions; one particular condition favored the dimer and the other favored the tetramer and has little to do with the species of the protein. If we conducted an even larger number of trials for the mouse Perk, we may eventually find a condition which could favour tetramer formation, and similarly with the human LD. We have added a statement in the solution structure analysis section to make this clear: "The ability to crystalize the proteins in different states are purely a result of the crystallization conditions favoring that particular state, and by chance we were able to capture both states in our crystallization experiments". These two states that we visualized in the crystal are exactly how they exist in solution as we have confirmed this by our SAXS analysis.

We would like to assure the reviewer that the structure has been built to a high standard, perfectly in line with quality criteria for measuring crystal structural data (see table1). Also, I have solved other structures before including the human Ire1 RNase and Kinase domain crystal structure (Ali et al EMBO J 2011). For data statistics please see table 1.

<u>In the same idea, SEC-MALS and analytical ultracentrifugation was only done with the luminal domain of human PERK. Is it possible to identify dimers and tetramers in solution of luminal domain of mouse PERK?</u>

Yes we now show that both human and mouse LD form dimer and tetramers in solution, and in a similar ratio $_{dimer}3{:}2_{tetramer}$ see figure 5A,B. This new data has greatly strengthened our manuscript!

<u>Although the authors evaluated the formation of tetramers in solution using analytical ultracentrifugation, it will be useful to evaluate if the tretrameric conformation is also present in vivo. Some experiments using crosslinking of proteins could be done to evaluate if these tetramers are in fact present on a cellular context and if the transition from dimeric to tetrameric states of human PERK is dependent of ER stress. This is a key experiment to provide.</u>

We have attempted to do this experiment, but it did not work. We believe that this is because Perk is a membrane protein and a cross-linking membrane protein in vivo is not really feasible. We have gone through the literature and have found other examples where people have observed LD tetramers in vitro (1, 2) without explicitly saying so or describing the function that we do: that is the tetramer is required for high efficiency phosphorylation. However, interestingly, there is a report that shows Ire1 Luminal domain, but not full length protein, forming tetramers by cross-linking in vivo (3), but we were unable to reproduce this with Perk. I think that this maybe due to our cellular technical ability not being as good (we are mainly structural and protein in vitro specialists) as the lab who observed LD tetramers in vivo, see figure 8 in ref (3). This important report has been referenced in the discussion as evidence for LD tetramer in vivo.

<u>In addition, the authors discuss the possibility that these dimeric and tetrameric states are part of a regulatory step in PERK activation. If so, Do the WT PERK and W165A or L388N varianst have the same affinity of interaction with BiP? Can this explain the differences in activation of PERK? Co-IP experiments could be performed to address this point</u>.

In our previous manuscript we presented, in the discussion, a possible model that suggests differences between activation maybe due to binding of BiP. We have now edited this model out, as we do not have data to suggest that this is the case. For interest, we pursued the reviewer's request, but we did so using MST interaction analysis. We conducted protein interaction analysis with the mutant, L388N, and BiP and compared to wild type Perk LD. We found no significant difference. In the revised manuscript we do not mention interaction with BiP. The discussion now focuses on our main point that the tetramer is important for higher efficiency phosphorylation, possibly by providing a sturdier platform than the dimer for phosphorylation to occur.

<u>In figure 6A, the authors generate a series of point mutations based in structural analysis of the tetramer subdomain W165A and L388N partially abolish the formation of tetramers. In addition, this phenotype correlates with less activation of PERK evaluated by phospho-PERK under ER stress conditions. Although these experiments suggest decreased activation, HT1080 cells have endogenous PERK so</u>

this can mask the real impact of W165A and L388N mutants in PERK signaling. A PERK KO background could be used if possible.

We have now redone the experiment in PERK-/- cells as the reviewer has asked. Furthermore we have analyzed 5 mutations within the tetramer interface both in vitro by AUC analysis, and in vivo measuring Perk phosphorylation and eIF2A phosphorylation (See Figure 6). We thank the reviewer for this excellent suggestion that has greatly improved the manuscript.

In addition, a more detailed analysis of PERK signaling should be evaluates with other parameters like eIF2α phosphorylation and ATF4 or CHOP expression.

We now not only measure Perk phosphorylation, but also measure eIF2A phosphorylation as well. We also tried to measure Chop levels but were unable to get a reliable signal at the right size (reviewer 2 wanted the gold standard eIF2a only, which we have done). However, we feel the point is clear using Perk-phosphorylation and eIf2a-phophosphorylation levels. Both these indicators reinforce the point that tetramer mutations have an effect of reducing Perk signaling.

If mouse PERK does not form tetramers, important controls can be done generating mutants of mouse PERK (W165A and L388N) and evaluate its activation. If this is the case, these mutants should not affect the activation of mouse PERK under ER stress conditions.

We show that both mouse and human Perk LD form dimers and tetramers in solution by AUC analysis in 3:2 ratio, and is confirmed by SAXS analysis.

IRE1a has been suggested that IRE1a can assembly into oligomers to form clusters (Korennykh et al, Nature, 2009 and Li et al, PNAS, 2010). Generation of PERK oligomers in vivo should be tested.

Our data shows the existence of dimers and tetramers for Perk LD, but not anything else. This does not mean that higher oligomers cannot form – it may form from a tetramer state. We just don't see them in our experiments. Studies showing oligomers of Ire1 are principally done in vivo only. We think that testing the existence of an oligomeric state in vivo that we do not see/or mention in any way in our paper would be unfair, particularly as the techniques used involve cell imaging/microscopy, and are techniques we have never used.

Reviewer 2

We appreciate the reviewers comments.

What is the level of conservation of the tetramerization interface between human and mouse PERK? In particular the tetramerization sub-element helix α2. Since mouse and human PERK are highly similar proteins, it is surprising that one forms a tetramer while the other does not. This discrepancy strongly hints that the tetramer arrangement may be a species-specific artifact of high protein concentrations, which is not uncommon.

In Fig2D we provide a sequence alignment of residues involved in the tetramer domain from: PERK human and mouse, and IRE1 human and mouse (we also

provide a full sequence alignment in the supplemental data, and a structural alignment). The fig shows that there is high level of conservation.
We now have added new data - and I believe this will really clarify the results - that show both human and mouse Perk LD can form dimers and tetramers, see Fig 5A.
We believe the difference in capturing these two states lies solely in the crystallization conditions; one particular condition favored the dimer and the other favored the tetramer and has little to do with the species of the protein. Or even the protein concentration, human LD protein was at a concentration of 5mg/ml for crystallization trials, whilst mouse LD was at 20mg/ml, over four times more concentrated than human protein LD, yet mouse LD formed dimers only, whilst human LD formed tetramer. If we conducted an even larger number of trials for the mouse perk we may eventually find a condition which would favour tetramer for mouse, and tetramer formation for human LD. We have added a statement in the solution structure analysis section to make this clear: “The ability to crystalize the proteins in different states are purely a result of the crystallization conditions favoring that particular state, and by chance we were able to capture both states in our crystallization experiments”.

The tetramerization interface has not been extensively validated by mutagenesis. Only two mutants W165A and L388N were tested and both do not completely abolish tetramer formation in vitro, nor PERK autophosphorylation in vivo. Additional mutants are required to validate the tetramerization model for PERK activation. The authors should consider introducing large bulky residues or charge repulsive substitutions into helix α2 to more aggressively perturb the tetramer interaction.

We have now characterized 5 mutations in vitro by AUC and also in Perk-/- cells. We see a significant shift towards dimer formation for all mutations in vitro, up to a high of 60% See Figure 6. This is mirrored in reduction in Phosphorylation in cells. The tetramer interface is predominantly comprised of hydrophobic residues, we have mutated the most conserved residues, which mainly consists of Leu to Asn. This mutation completely reverses a highly hydrophobic residue for a completely hydrophilic residue of similar size – therefore purely targeting/distrupting the hydrophobic nature of the tetramer interface.

We also attempted to introduce bulky mutations such as Leu to phe as the reviewer suggested, but this protein was not soluble to test in vitro and hence we did not continue with it.

However, the reviewer has asked for abolishment mutations, but this is not biologically feasible. It is widely accepted that the dimer is sufficient for trans auto phosphorylation to occur (2, 4-8) and since we do not target the dimer interface we will never achieve abolishment of phosphorylation with the tetramer. What we do see is a reduction of phosphorylation upon mutation of the tetramer interface, which suggests that the tetramer is required for high efficiency phosphorylation (see discussion), possibly by providing a sturdier platform for phosphorylation to take place.

3) In Figure 6 the authors seek to address whether PERK tetramerization influences PERK kinase domain activation ? Since PERK kinase domain functions as a back-to-back dimer, perhaps tetramerization is important for enabling one PERK dimer to phosphorylate another PERK dimer during the autophosphorylation reaction. The authors show a modest decrease in PERK pThr980 levels for a tetramer interface mutant but total PERK levels were also decreased so one cannot make a solid conclusion. The authors did not measure pSer51 eIF2α levels, which is the gold

standard for assessing UPR activation. Stronger mutants and equal levels of protein expression are required to make a compelling case that the tetramer configuration shown in the crystal structure is biologically relevant and not simply an artifact.

We have now completely redone the experiment, using 5 strong hydrophobic to polar reversal mutations, initially characterized in vitro, that show a reduction as compared to the WT of up to 60%. Furthermore, we have done the experiments in Perk-/- cells and now measure the gold standard eIf2a as the reviewer has asked. We see a reduction in Perk and eIF2a levels. Since the dimer is sufficient for phosphorylation to occur, we believe the mutations are really strong and emphasis the point that the tetramer is required for higher efficiency phosphorylation.

4) The authors argue that the PERK luminal domain functions as a constitutive dimer that transitions into a tetramer for activation (Figure7). Is there any evidence of a constitutive PERK dimer in vivo? Is there evidence that BiP preferentially binds luminal domain dimers vs. monomers? Does BiP binding inhibit tetramerization? More experimental data is required to support the proposed model. Currently the proposed model is highly speculative.

We agree with the reviewer. We have removed the word constitutive and replaced it with the word “stable” dimer. The word “stable” has been used by many authors before to describe the dimer arrangement. Also, the BiP binding model was highly speculative, and hence we originally put in the discussion. However, we have now removed this model and do not mention the interaction with BiP. We only show a model consistent with our data that the tetramer is required for higher efficiency phosphorylation.

Does the oligomerization surface of yeast IRE1 overlap with the tetramerization interface of human PERK? Oligomerization of yeast IRE1 luminal domain was proposed to be induced by direct binding of unfolded proteins. Might PERK tetramerization allow for direct binding of unfolded proteins?

The key residue that was mutated in the yeast structure Trp 426, which was used as verification of interface 2 is not present in the Perk tetramer structure. There is little overlap between the tetramer interface and the interface 2, suggesting that they may be able to form oligomers, possibly from a tetramer state. Since we do not see or mention oligomer formation in the paper, we do not discuss this further.

We have now revised the discussion and hence we do not speculate on BiP or unfolded protein binding to luminal domains. The discussion focuses on the tetramer being required for higher efficiency phosphorylation, consistent with the data presented.

Reviewer 3

We thank and greatly appreciate this reviewer’s positive comments!

This manuscript describes a structural study of the luminal domain of the endoplasmic reticulum stress sensing protein kinase, Perk, which plays a key role in the signaling processes that accompany the unfolded protein response (UPR). The

structural studies feed into a series of downstream biophysical and cellular studies that test the implications of the crystal structures, and suggest a role for tetramer formation in Perk function in the UPR. Overall the manuscript is very clearly written, the figures are effective, and for the most part the conclusions are well supported by the data presented.

The only serious shortcoming occurs in the downstream experiments where the authors engineer mutations designed to prevent tetramer formation and validate the effect of these in vitro by AUC and SAXS. They then measure consequences of these mutations on Perk phosphorylation (presumably auto-phosphorylation but not clearly stated as such) in cells following conventional UPR induction by tunicamycin. Consistent with their hypothesis that tetramer formation is functionally important the western blots show decreased signal for phosphorylated Perk at each time point for the mutants compared to the wild-type. However, the only control presented is a GAPDH loading control, which only assures that more or less equal numbers of cells were analysed in each lane.

It is essential before this data can be used, that the authors also blot for total Perk and show this alongside the phospho-Perk signal. Without this control they cannot reliably conclude that the mutations impair the mechanism of Perk activation and signalling rather than simply destabilize the protein. It would be nice, though perhaps not essential, if they also provided fluorescence microscopy data showing that the ER localization of the mutant protein is not affected by the mutations.

The authors provide a useful discussion of the mechanistic implications of their model and reasonably speculate on the basis of conservation that it might also occur in Ire1 - at least in mammalian species. It would be interesting to see some discussion of the implications of this for unraveling the complicated and somewhat controversial models (especially for Ire1) of how luminal association of these transmembrane proteins promotes association and activation of their cytoplasmic kinase domains. The senior author did provide one of the more important papers in that controversy a few years ago, so it would be good to read how they bring this new luminal tetramerisation model to bear on this issue.

We have now completely redone the in vivo analysis in Perk -/- cells and measured both Perk and eIF2A phosphorylation. Also, we measure total perk levels by using a myc tagged Perk, as the reviewer has suggested for the important control. Also, we measure 5 mutations to really reinforce the tetramer interface.

We have also revised the discussion and focused on our point that the tetramer is important for higher efficiency phosphorylation, consistent with our data.

Reference

1. Gardner BM & Walter P (2011) Unfolded proteins are Ire1-activating ligands that directly induce the unfolded protein response. *Science* 333(6051):1891-1894.
2. Zhou J*, et al.* (2006) The crystal structure of human IRE1 luminal domain reveals a conserved dimerization interface required for activation of the unfolded protein response. *Proceedings of the National Academy of Sciences of the United States of America* 103(39):14343-14348.

3. Liu CY (2002) The Protein Kinase/Endoribonuclease IRE1alpha That Signals the Unfolded Protein Response Has a Luminal N-terminal Ligand-independent Dimerization Domain. *Journal of Biological Chemistry* 277(21):18346-18356.
4. Walter P & Ron D (2011) The unfolded protein response: from stress pathway to homeostatic regulation. *Science* 334(6059):1081-1086.
5. Ali MMU*, et al.* (2011) Structure of the Ire1 autophosphorylation complex and implications for the unfolded protein response. *The EMBO journal* 30(5):894-905.
6. Ma K, Vattem KM, & Wek RC (2002) Dimerization and release of molecular chaperone inhibition facilitate activation of eukaryotic initiation factor-2 kinase in response to endoplasmic reticulum stress. *J Biol Chem* 277(21):18728-18735.
7. Liu CY, Schroder M, & Kaufman RJ (2000) Ligand-independent dimerization activates the stress response kinases IRE1 and PERK in the lumen of the endoplasmic reticulum. *J Biol Chem* 275(32):24881-24885.
8. Shamu CE & P. W (1996) Oligomerization and phosphorylation of the Ire1p kinase during intracellular signaling from the endoplasmic reticulum to the nucleus. *EMBO J* 15:3028-3039.

2nd Editorial Decision 17 March 2015

Thank you for your patience during our re-consideration of your manuscript on PERK luminal domain structure and oligomerization. I have now heard back from the original referees 1 and 2, and I am pleased to inform you that both of them consider the most critical concerns from the initial round of review adequately addressed. Following a few additional minor modifications, we shall therefore be happy to accept the study for publication in The EMBO Journal.

Regarding the remaining referee points, please amend the manuscript with the controls requested by referee 2. Please also consider referee 1's point 1 regarding more detailed discussion of mechanistic implications, maybe also in the light of your recent eLife article (Carrara et al) and possibly mentioning/showing the negative BiP interaction as a supplementary figure. On the other hand, it will not be necessary to obtain further data on effects on downstream signaling (ref 1 point 2 - I agree that assessment of the 'gold standard' eIF2alpha phosphorylation is sufficient within the scope of this work). Furthermore, I also appreciate the difficulties with in vivo demonstration of transient PERK LD tetramerization (ref 1 point 3), where even crosslinking of a soluble domain (as in Liu et al 2002) may by itself not be too informative, and more dedicated approaches such as FRET sensors would appear better suited for future projects.

When re-revising the manuscript, please do consider also the following editorial points:

- Please provide us (in your resubmission cover letter) with 2-5 short one-sentence 'bullet points', containing brief factual statements that summarize key aspects of the paper - they will accompany the online version of the article as part of a 'synopsis'. Please see the latest research articles on our website (emboj.embopress.org) for examples - I am happy to offer further guidance on this if necessary.

- Please make sure to include PDB accession numbers in the final manuscript version.

- I would encourage you to replace 'Perk' with 'PERK' throughout the manuscript (unless you may want to retain 'Perk' in those instance referring specifically to the mouse enzyme), in line with nomenclature of the human protein and to better differentiate this as an acronym/abbreviation.

- I would suggest to modify the title slightly to "Crystal structures reveal transient PERK luminal domain tetramerization in ER stress signaling". This would make it more concise and easily understandable, while at the same time certainly implicating that tetramerization is 'important'.

- Furthermore, it may be beneficial to slightly edit the abstract to facilitate readability and make it easily accessible also to non-experts, as follows:

"Stress caused by accumulation of misfolded proteins within the endoplasmic reticulum (ER) elicits a cellular unfolded protein response (UPR) aimed at maintaining protein folding capacity. PERK, a key upstream component, recognizes ER stress via its luminal sensor domain, but the molecular events that lead to UPR activation remain unclear. Here, we describe the crystal structures of mammalian PERK luminal domains captured in dimeric state as well as in a novel tetrameric state. Small angle X-ray scattering analysis (SAXS) supports the existence of both crystal structures also in solution. The salient feature of the tetramer interface, a helix swapped between dimers, implies transient association. Moreover, interface mutations that disrupt tetramer formation in vitro reduce phosphorylation of PERK and its target eIF2 in cells. These results suggest that transient conversion from dimeric to tetrameric state may be a key regulatory step in UPR activation."

- Finally, in order to make the primary data behind the (somewhat over-contrasted/bright) blots in Figure 6B panels more accessible and more directly represented, I would kindly ask you to include figure source data for these gels/blots. A PDF file containing the original uncropped and unprocessed scans including only basic annotation as well as molecular weight markers can be uploaded upon resubmission selecting "Figure Source Data" as object type, and would be linked as such to the respective figure in the online publication of your article.

At this stage, I am therefore returning the manuscript to you for an ultimate round of minor

modification. Once we will have received your final version, we should then be in a position to swiftly proceed with its formal acceptance and publication.

———

REFEREE REPORTS:

Referee #1:

The revised manuscript by Carrara and colleagues solved the crystal structure of human and mouse PERK capturing two states of organization: dimers and tetramers. Importantly, in this resubmission the authors were able to demonstrate these oligomeric forms of PERK in human and mouse. In addition, in mutants of PERK that forms tetramers in a less extend, reduced PERK phosphorylation and eIF2 is observed. Overall with the new data the manuscript has clearly improved in terms of consistency and in vitro analysis. Most of our concerns were solved; however there are some points still missing, especially related to in vivo validation in simple cell culture models. This is an excellent paper with important and high quality data. My concerns are mostly focus in making sure that tetramers have functional relevance in living cells and that they are observed in living cells.

Specific comments

1) The authors also mentioned as data not shown that using MST interaction assay no changes are observed between mutant L388N and WT in terms of interactions with BiP. These data should be added to the manuscript.
In addition, a more detailed discussion should be included in order to explain the relevance of the tetramers for PERK activation.

2) As requested, the authors have performed experiments using L388N, W165A, L395N, L397N and A378N PERK mutants in PERK KO background. In this experimental settings, there are less PERK and eIF2 phosphorylation under ER stress conditions. These results are highly important and should be confirmed with other readouts to have a stronger message. Some downstream signaling outputs like ATF4, CHOP, BiP, or GADD34 could be easily evaluated at the protein or mRNA level. This data will improve the cellular biology behind the in vitro experiments already performed and might help to elucidate if dimers vs tetramers can impact differentially the signaling outputs downstream of PERK activation

3) Finally, the authors evaluated the formation of dimers and tetramers in solution using analytical ultracentrifugation. As requested before, it is absolutely necessary to observe if the tetrameric conformation is also present in a cellular model. Again, crosslinking experiment, non-denaturing electrophoresis, etc could be done easily to address this point with the PERK KO cells that were reconstituted with different constructs. We know this is challenging but other labs have reported assays to do this.

Referee #2:

The authors describe the crystal structure of the luminal domain of the unfolded protein response (UPR) effector human PERK in a novel tetramer configuration. Tetramerization involves a helix swap mechanism, between dimers that themselves adopt a previously observed dimer conformation by the luminal domains of human and yeast Ire1. Yeast Ire1 dimers but not human Ire1 dimers also assemble into a higher order filament arrangement but one that is totally different from the tetramer

arrangement shown here for human and mouse PERK.

Based on new and revised data presented, I believe that the authors have sufficient data to warrant publication in EMBO. Minor controls are required before publication.

Key New data presented:

1) The authors previously showed that human PERK formed tetramers (dimers of dimers) while mouse PERK only formed dimers in their crystal structures without convincingly validating these higher-order states in solution. Since mouse and human PERK are highly similar proteins, it was surprising that one formed a tetramer while the other did not. This discrepancy strongly hinted that the tetramer arrangement of human PERK may have been a species-specific artifact of high protein concentrations during crystallization. However, the authors now show convincing evidence by AUC and SAXS analyses that both mouse and human PERK form tetramers in solution.

2) The authors previously showed two mutants W165A and L388N that reduced tetramer formation in vitro and PERK auto-phosphorylation in ER-stressed cells. They now include three additional mutants L395N, L397N, A378N that show the same effects. Moreover, the authors now test for both PERK auto-phosphorylation and eIF2 phosphorylation.

The mutants that the authors have generated at the PERK tetramer interface do not completely abolish tetramerization by AUC. However, the reduction of approximately 50%-60% of the tetramer species is enough to exert a significant effect when these mutants were introduced into ER-stressed cells. Under ER stress conditions, PERK mutants show significant reduction in both Thr980 auto-phosphorylation and Ser51 eIF2 phosphorylation, as compared to wild type PERK protein. The observation that tetramerization mutants show decreased autophosphorylation supports a model whereby interdimer autophosphorylation is reduced at the PERK activation step, upstream of eIF2 phosphorylation.

Key controls that are currently lacking but are easy to provide include:

Figure 6B
The authors are missing a blot showing that total eIF2 levels are similar and hence comparable. Also please add a protein size ladder and label which antibody was used in the Western blots.

2nd Revision - authors' response 08 April 2015

Referee 1

1) The authors also mentioned as data not shown that using MST interaction assay no changes are observed between mutant L388N and WT in terms of interactions with BiP. These data should be added to the manuscript.
In addition, a more detailed discussion should be included in order to explain the relevance of the tetramers for PERK activation

We have now revised the discussion adding more detail to the role of tetramers in PERK activation. We have also discussed the current results in the context of our recent eLife paper (Carrara et al 2015) where we describe a novel allosteric mechanism for UPR induction. The current work fits in excellently with the new proposed mechanism. Furthermore, we mention the MST binding data between WT and mutant PERK with BiP, and we have included the data as supplementary figure 5.

Referee 2

© European Molecular Biology Organization

Figure 6B: The authors are missing a blot showing that total eIF2α levels are similar and hence comparable. Also please add a protein size ladder and label which antibody was used in the Western blots.

Done! We have now added protein size markers and have made it clear which antibodies were used. Full details of concentration, manufacturer, and the protocol used for each antibody are listed within the material and methods section. Furthermore, we have presented the full western blots for both phospho-eIF2α and phospho-PERK with markers as a source data file.

Acceptance letter 13 April 2015

Thank you for submitting your final revised manuscript for our consideration. I am pleased to inform you that we have now accepted it for publication in The EMBO Journal.
