# Supplementary figures and images for: Crystal structures reveal transient PERK luminal domain tetramerization in endoplasmic reticulum stress signaling
Source: EMBO J. 2015 Apr 29;34(11):1589–600. doi: 10.15252/embj.201489183 (PMC4474532; doi:10.15252/embj.201489183)

### Phospho-PERK

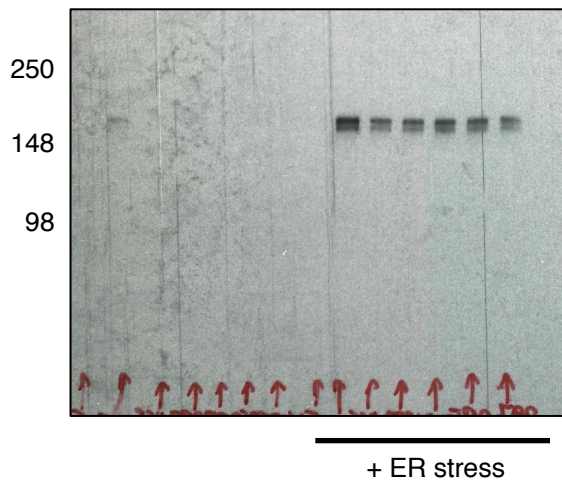

### Phospho-eIF2 $\alpha$

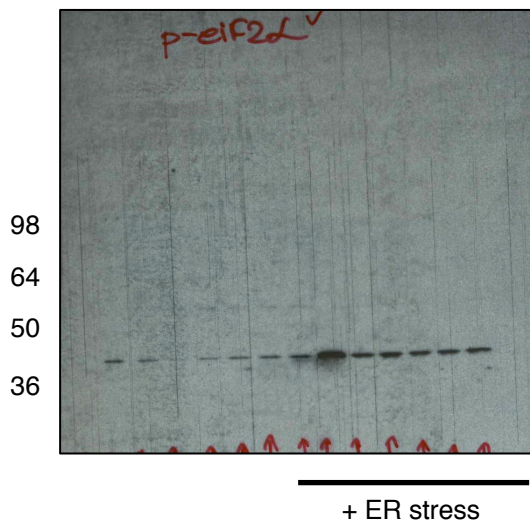

Supplement: Supplementary file 3 [file embj0034-1589-sd3.pdf]
